# Supplementary material for: Metal-ligand dual-site single-atom nanozyme mimicking urate oxidase with high substrates specificity
Source: Nat Commun. 2024 Jul 8;15:5705. doi: 10.1038/s41467-024-50123-4 (PMC11231224; doi:10.1038/s41467-024-50123-4)
Supplement: Supplementary file 1 — Supplementary Information [file 41467_2024_50123_MOESM1_ESM.pdf]

Supplementary information

Metal-ligand dual-site single-atom nanozyme mimicking urate oxidase with high substrates specificity

Kaiyuan Wang,[1] Qing Hong,[1] Caixia Zhu,[1]Yuan Xu,[1] Wang Li,[1] Ying Wang,[1] Wenhao Chen,[1] Xiang Gu,[1] Xinghua Chen,[1] Yanfeng Fang,[1] Yanfei Shen,* [2] Songqin Liu,[1] Yuanjian Zhang*[1, 3]

[1] Jiangsu Engineering Research Center for Carbon-Rich Materials and Devices, Jiangsu Province Hi-Tech Key Laboratory for Bio-Medical Research, School of Chemistry and Chemical Engineering, Nanjing 211189, China.
[2]Medical School, Southeast University, Nanjing 210009, China.
[3]Department of Oncology, Zhongda Hospital, Southeast University, Nanjing 210009, China.

Email: Yuanjian.Zhang@seu.edu.cn (Y.Z.); Yanfei.Shen@seu.edu.cn (Y.S.)

Table of Contents

Supplementary Methods........ 3-6
Supplementary Notes........ 7-9
Supplementary Fig. 1........ 10
Supplementary Fig. 2........ 11
Supplementary Fig. 3........ 12
Supplementary Fig. 4........ 13
Supplementary Fig. 5........ 14
Supplementary Fig. 6........ 15
Supplementary Fig. 7........ 16
Supplementary Fig. 8........ 17
Supplementary Fig. 9........ 18
Supplementary Fig. 10........ 19
Supplementary Fig. 11........ 20
Supplementary Fig. 12........ 21
Supplementary Fig. 13........ 22
Supplementary Fig. 14........ 23
Supplementary Fig. 15........ 24
Supplementary Fig. 16........ 25
Supplementary Fig. 17........ 26
Supplementary Fig. 18........ 27
Supplementary Fig. 19........ 28
Supplementary Fig. 20........ 29
Supplementary Fig. 21........ 30
Supplementary Fig. 22........ 31
Supplementary Fig. 23........ 32
Supplementary Fig. 24........ 33
Supplementary Fig. 25........ 34
Supplementary Fig. 26........ 35
Supplementary Fig. 27........ 36
Supplementary Fig. 28........ 34
Supplementary Fig. 29........ 38
Supplementary Fig. 30........ 39
Supplementary Fig. 31........ 40
Supplementary Fig. 32........ 41
Supplementary Fig. 33........ 42
Supplementary Fig. 34........ 43
Supplementary Fig. 35........ 44
Supplementary Fig. 36........ 45
Supplementary Fig. 37........ 46
Supplementary Fig. 38........ 47
Supplementary Fig. 39........ 48
Supplementary Fig. 40........ 49

Supplementary Fig. 41.. 50
Supplementary Fig. 42.. 51
Supplementary Fig. 43.. 52
Supplementary Fig. 44.. 53
Supplementary Fig. 45.. 54
Supplementary Fig. 46.. 55
Supplementary Fig. 47.. 56
Supplementary Fig. 48.. 57
Supplementary Fig. 49.. 58
Supplementary Fig. 50.. 59
Supplementary Fig. 51.. 50
Supplementary Fig. 52.. 61
Supplementary Fig. 53.. 62
Supplementary Fig. 54.. 63
Supplementary Fig. 55.. 64
Supplementary Table 1.. 65
Supplementary Table 2.. 66
Supplementary Table 3.. 67
Supplementary Table 4.. 68
Supplementary Table 5.. 69
Supplementary References.. 70

Supplementary Methods

Chemicals

Nickel (II) chloride hexahydrate ($NiCl_2 \cdot 6H_2O$), cobalt (II) chloride hexahydrate ($CoCl_2 \cdot 6H_2O$), ammonia, acetone, uric acid (UA), o-phenylenediamine (OPD), glucose (Glu), sodium hydroxide (NaOH), potassium hydroxide (KOH) and potassium thiocyanate (KSCN) were purchased from Sinopharm Chemical Reagent Co., Ltd. (China). HRP, Pt/C (20%), 3,3',5,5'-tetramethylbenzidine (TMB) and 2,2'-azinobis-(3-ethylbenzthiazoline-6-sulphonate) (ABTS) were purchased from Sigma-Aldrich. 2,4-dichlorophenol (2,4-DP), epinephrine (E), 4-aminoantipyrine (4-AP), dopamine (DA) and 3,5-ditert-butyl catechol (DTBC) were purchased from Aladdin Chemistry Co., Ltd. (China). 3,3'-diaminobenzidine tetrahydrochloride (DAB·4HCl), 1,2,4,5-benzenetetramine tetrahydrochloride (BTA·4HCl), heavy-oxygen water ($H_2{}^{18}O$), uricase (from arthrobacter protophormiae, ≥20 U/mg) were purchased from Shanghai Macklin Biochemical Co., Ltd. (China). Ketjenblack EC600J carbon black (CB) was purchased from Akzo Nobel N.V. (Netherlands). All other chemicals were used without further purification unless otherwise specified. Ultrapure water (18.2 MΩ cm) used in all the experiments was obtained from a Direct-Q 3 UV pure water purification system (Millipore, USA).

Characterization. Fourier transform infrared spectra (FT-IR) were recorded with a Nicolet iS20 FT-IR spectrometer (Thermo Fisher, USA) with the KBr tableting. NMR spectra were conducted on AVANCE III 400 MHz WB solid-state NMR spectrometer (Bruker, Germany). Transmission electron microscopy (TEM) images were collected by Talos F200X transmission electron microscopy (Thermo Fisher, USA). Scanning electron microscopy (SEM) images were obtained from a Navo Nano SEM450 scanning electron microscopy (FEI, America). High-angle annular dark field scanning transmission electron microscopy (HAADF-STEM) images were performed by JEM-ARM300F GRAND ARM (JEOL, Japan). The ESCALAB 250Xi electron spectrometer

(Thermo Fisher, USA) was employed as the X-ray photoelectron spectrum (XPS) characterization with the peak of C1*s* (284.8 eV) as the reference for calibration. The Ni concentration was measured by Agilent 7800 inductively coupled plasma mass spectrometry (ICP-MS) (Agilent, Singapore). UV-vis absorption spectra were measured using the Cary100 UV-vis spectrophotometer (Agilent, Singapore). Electrospray ionization mass spectra (ESI-MS) were measured by LCMS6120 liquid phase quadrupole mass spectrometer (Agilent, Singapore). Thermal gravimetric analysis (TGA) was conducted in N_2 atmospheres (100 mL min^{-1}) with a heating rate of 10 °C min^{-1} by SDT-Q600 instrument (TA, USA). The combustion elemental analysis was investigated by a Vario MICRO Cube (Elementar, German). The electrochemical measurements were performed by a CHI700E workstation (CHI, USA) and RRDE-3A rotating ring-disk electrodes (ALS, Japan). N_2 adsorption-desorption isotherms (77 K) were collected by an autosorb iQ (Quantachrome, USA), and samples were degassed for 12 h at 150 °C under reduced pressure before measurement. Oxygen temperature programmed desorption (O_2-TPD) was measured by AutoChem II 2920 (Micromeritics, USA). Electron spin resonance (ESR) spectra were obtained from the EMXPlus spectrometer (Bruker, Germany) at room temperature.

Isotope labeling experiment

In $H_2{}^{18}O$, Ni-DAB (50.0 μg mL^{-1}) and UA (0.1 mM, dissolved in 10mM Li_2CO_3) were mixed at 25 °C for 30 min. The product was detected by ESI-MS after filtration.

TGA cycle tests

3 mg of fully dried Ni-DAB was tested in a thermogravimetric crucible in the normal procedure (10 °C min^{-1}, RT to 150 °C, N_2). After the test, wait for the sample to cool to RT, remove it, expose the sample to dry air for 30 min to re-adsorb the gas, and then perform the TGA test using the same procedure. The measurement was repeated three times in this manner.

Monitoring $\cdot O_2^-$ species by EPR.

DMPO (5 μL), Ni-DAB (10 μL, 5 mg mL^{-1}), and UA (10 μL, 10 mM) were added into an air-saturated methanol solution (75 μL) and incubated for 5 min at RT. Then, the mixture was transferred into a capillary tube for EPR measurements.

EPR measurements of electron transfer

Ni-DAB (10 mg) and substrates (3 mg) were added into a N_2-saturated buffer solution (100 μL) and sonicated for 30 min at RT. Then, the mixture was transferred into a capillary tube for EPR measurements.

Electrochemical tests

The ORR activities of Ni-DAB/C and commercial Pt/C (platinum, 20% on carbon black) were evaluated in a standard three-electrode glass cell on a CHI700E workstation (CHI, USA). A rotating disk glassy carbon electrode (RDE), modified with an electrocatalyst, was used as the working electrode. A Pt wire and mercury oxide electrode were used as the counter and reference electrodes, respectively. The electrolyte (0.1 M KOH in water) was freshly prepared during the electrochemical investigation. The procedure for working electrode modification was as follows. The electrocatalyst suspension (12 μL) in water (5 mg mL^{-1}) was cast on the pre-polished surface of the RDE (3 mm diameter), resulting in a catalyst loading of 849 μg cm^{-2}. The electrode was then allowed to dry at 37 °C for 1h. Next, 2 μL of PVDF (1 mg ml^{-1}) was cast on the surface of the electrode. The electrode was then dried again at 37 °C for 6h. For the ORR test, 0.1 M KOH (pH=13) was purged with high-purity O_2 gas for 30 min, and thereafter, the electrolyte was protected by O_2 flow. The ORR activity was measured by linear sweep voltammetry (LSV) using a rotating electrode system (RRDE-3A, BAS, Japan). LSV was recorded at a scan rate of 10 mV s^{-1} in the potential range of 0.2 to 1.0 V vs. RHE (E(RHE)=0.098+E+0.059×pH). During the experiment, various concentrations of the KSCN solution were added to the electrolyte to test the effect of SCN^- on the catalyst.

XAS measurement

Ni K-edge analysis was performed with Si (111) crystal monochromators at the BL14W1 beamlines at the Shanghai Synchrotron Radiation Facility (SSRF, Shanghai, China). Before the analysis at the beamline, the samples were pressed into thin sheets of 1 cm diameter and sealed using Kapton tape film. XAFS spectra were recorded at RT using a 4-channel Silicon Drift Detector (SDD, Bruker 5040). Ni K-edge extended X-ray absorption fine structure (EXAFS) spectra were recorded in transmission mode. Negligible changes in the line shape and peak position of the Ni K-edge XANES spectra were observed between the two scans obtained for a specific sample. The XAFS spectra of these standard samples (NiPc, Ni_2O_3, and Ni foil) were recorded in the transmission mode. The spectra were processed and analyzed using the software codes Athena and Artemis. The photoemission endstation at the BL10B beamline of the NSRL provides XAS beam times for the O K-edge analysis.

Computational details

The theoretical modellings in this study were performed with DMol3 code at the level of spin-unrestricted DFT[1, 2]. The double numerical basis set including popularization function (DNP), generalized gradient-corrected Perdew-Burke-Ernzerhof functional and an orbital cutoff of 5.0 Å was used for all calculations[3]. The solvation effect is accounted with a conductor-like screening model (COSMO). For geometry optimization, the tolerances of energy and force are 1×10^{-6} Ha and 0.002 Ha/Å, and the maximum displacement is 5×10^{-3}Å, respectively. Due to the 1D property of BTA-like structures, the Monkhorst-Pack k-point mesh was 4×1×1. The free energy diagrams were calculated using the method introduced in the Supplementary Information. The zero-point energies and entropies were computed from the partial hessian vibrational analysis, in which only the vibrational modes of the adsorbed species were calculated explicitly[4].

Supplementary Notes

Supplementary Note 1. The high-resolution N 1*s* XPS spectra in Supplementary Fig. 6a exhibited three main peaks, which were attributed to Ni-N (398.5 eV), C=N (399.3 eV), and C-N (400.6 eV). The high-resolution C 1*s* spectra of Ni-DAB in Supplementary Fig. 6b were deconvoluted into five types: C-C (284.8 eV), C-N (285.6 eV), C=N (286.3 eV), C-O (287.2 eV), and C=O (289.1 eV). Moreover, the high-resolution O 1*s* spectra of Ni-DAB in Supplementary Fig. 6c were assignable to adsorbed oxygen (532.1eV), C=O (533.2 eV), and C-O (534.6 eV).

Supplementary Note 2. The Cl element has been reported to serve as an axial ligand for Ni SACs and regulate catalytic activity[5]. Therefore, the models of Cl in the axial position of Ni were calculated. After structural optimization, it was found that the distances between Ni and Cl were too far (2.530 Å for single Cl atom absorption, 2.767/2.760 Å for two Cl atoms absorption) to be coordinated (Supplementary Fig. 9).

Supplementary Note 3. The first principle of molecular dynamics (MD) was used to imitate the stable configuration of Ni-DAB. A piece of Ni-DAB chain with two DAB molecules was used as the model. After 500 steps of MD at 300K, the configuration of Ni-DAB largely remained the initial configuration. The final frame was extracted and optimized, which gave the same configuration as the initial one (Supplementary Fig. 12). The long chain Ni-DAB was also built and optimized with the DFT method, which also showed a similar dihedral angle to the models. By the way, the model also has similar configurations to the ones reported in the literature[6, 7].

Supplementary Note 4. According to previous reports[8, 9], the amount of base added during preparation played a critical role in determining the coordination of polymer growth. The TEM images showed that the particle size of Ni-DAB improved progressively with increasing amounts of base (Supplementary Fig. 24). The high

amount of base may lead to a fast coordinated reaction and easy aggregation of products[9, 10]. Elemental analysis showed that Ni-DAB(11) (11 represents the volume of ammonia in mL added during the synthesis) with the largest particle size demonstrated the ligand to metal ratio of 1.5:1 (Supplementary Table 2). It had the lowest activity with a low metal content. Ni-DAB(3) had the highest activity and were explored in this study, unless otherwise specified. Ni-DAB(1) had a low aggregation due to an insufficient amount of base (Supplementary Fig. 25).

Supplementary Note 5. In the TGA curves (Supplementary Fig. 45b), Ni-DAB showed much higher thermal stability than DAB. The weight loss of Ni-DAB at about 350 °C could be ascribed to the defects (e.g., non-coordination sites, or free ligands). It should be noted that the Ni-DAB had no weight loss under 150 °C, while DAB does not; this loss could be ascribed to the adsorbed gas.

Supplementary Note 6. Fig. 3a showed that when the substrate transfers electrons to Ni-DAB, the EPR spectra intensity of Ni-DAB was enhanced. As for OPD, Glu, and 2,4-DP, no noticeable changes in the EPR spectra of Ni-DAB were noted, indicating the transfer of electron from these substrates to Ni-DAB was negligible (Supplementary Fig. 51). For ABTS, Supplementary Fig. 52 showed that the ABTS molecule has a strong EPR signal. After the addition of ABTS to Ni-DAB, the EPR signal of ABTS significantly decreased, while that of Ni-DAB was increased. This explained that there was a strong reaction and charge transfer from ABTS to Ni-DAB, consistent with the calculated results.

Supplementary Note 7. As controls, the calculation that UA oxidation and ORR catalyzed by the same Ni site (Ni single-site) on Ni-DAB was supplemented. Interestingly, the energy profile showed that this process is quite energetically favorable (Supplementary Fig. 53). For comparison, the calculation that UA oxidation and ORR catalyzed by the different Ni sites (Ni-Ni dual-site) on Ni-DAB was also performed. This time, there is an energy increase of 0.35 eV for the step of O_2→*OOH, indicating

the poor to activate O_2. These results suggested that the Ni center with the axial ligand can facilitate the ORR on it. However, we think such a single metal site catalyzed process is not dominant in the practical situation. Due to the high affinity of the Ni center for UA, the Ni center will be occupied by two UA molecules on either side of Ni-DAB (-0.53 eV and -0.55 eV) and O_2 cannot access the Ni center due to steric hindrance (Supplementary Fig. 54). In contrast, such UA occupation has no steric effect on the beta C site of Ni-DAB, which can provide reliable ORR site during reactions.

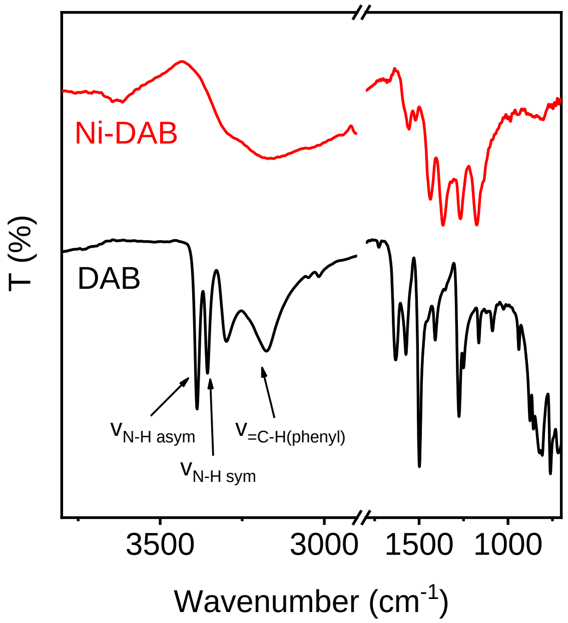

Supplementary Fig. 1. FTIR spectra of Ni-DAB and DAB.

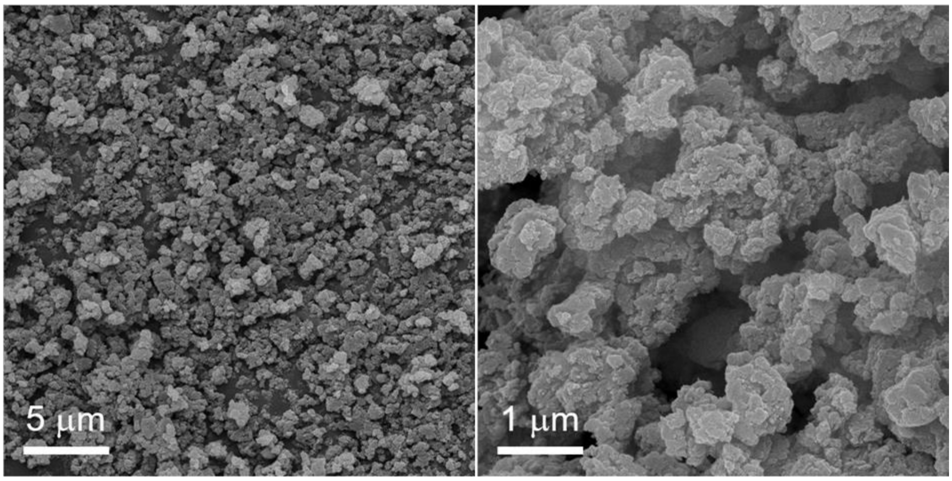

Supplementary Fig. 2. SEM images of Ni-DAB. Experiment was repeated 3 times independently with similar results.

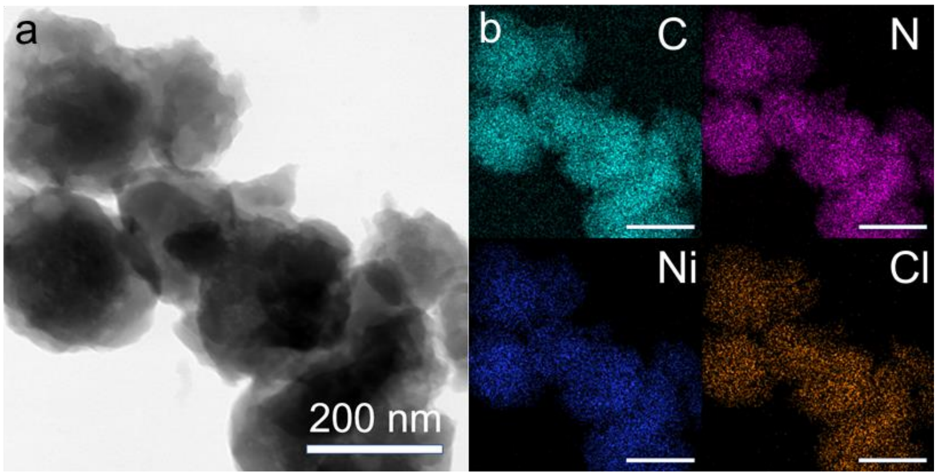

Supplementary Fig. 3. a, TEM image of Ni-DAB. **b**, and corresponding TEM-EDS elemental mapping images, showing the uniformly distributed C, N, Ni, and Cl elements in Ni-DAB. Scale bars: 200nm. Experiment was repeated 3 times independently with similar results.

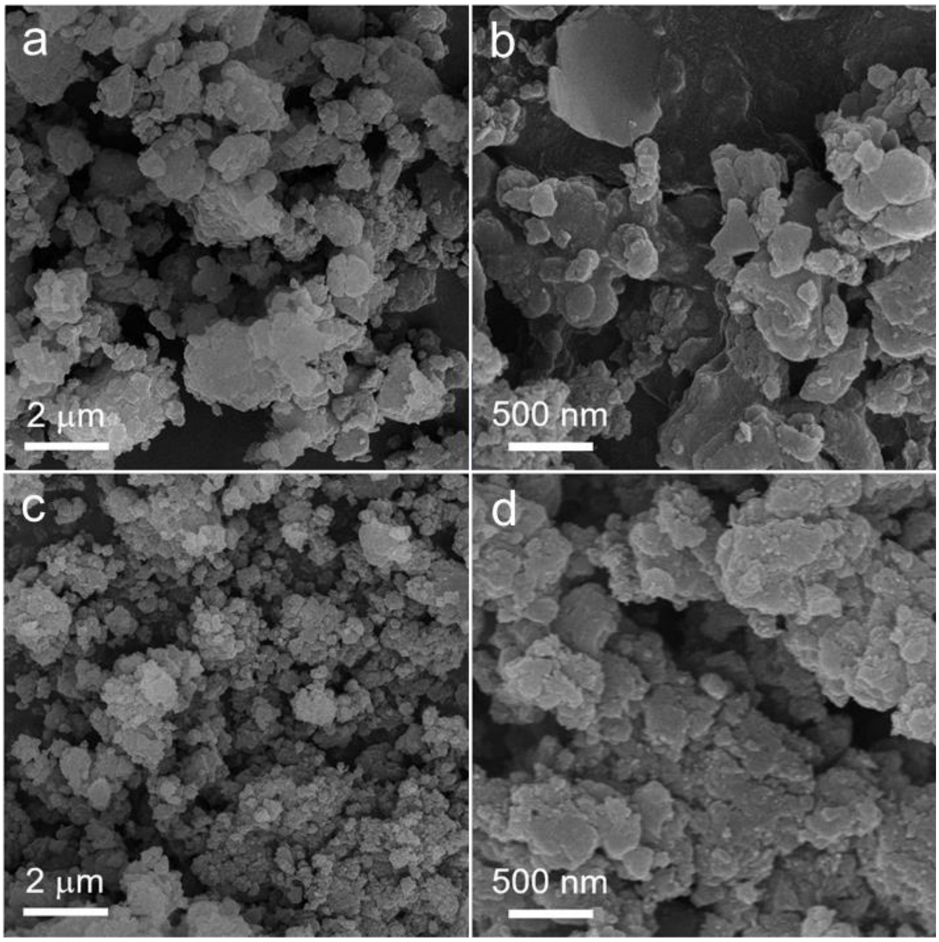

Supplementary Fig. 4. SEM images of control samples. a,b, Co-DAB. **c**,**d,** Ni-BTA. Experiment was repeated 3 times independently with similar results.

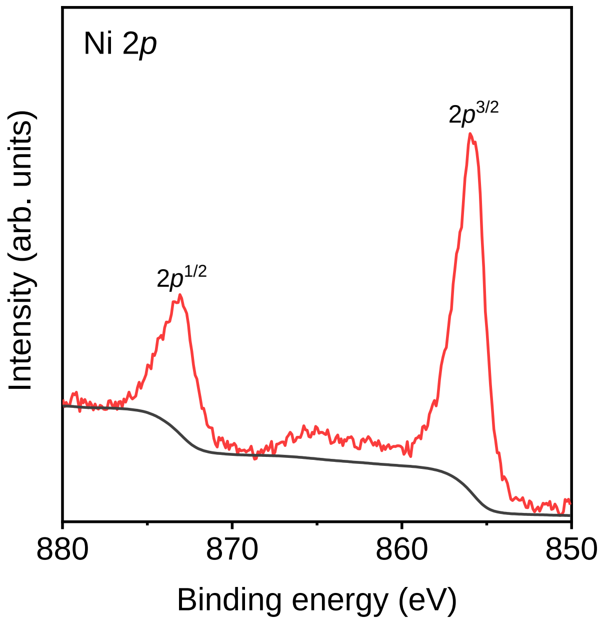

Supplementary Fig. 5. High-resolution Ni 2*p* XPS spectrum of Ni-DAB. The binding energy of Ni $2p^{3/2}$ in Ni-DAB was ~855.9 eV, which was ascribed to the Ni^{2+}. No peaks belonging to the metallic Ni^0 (~853.5 eV) or Ni^{4+} (~858 eV) were found[11]. "arb. units" refers to arbitrary units.

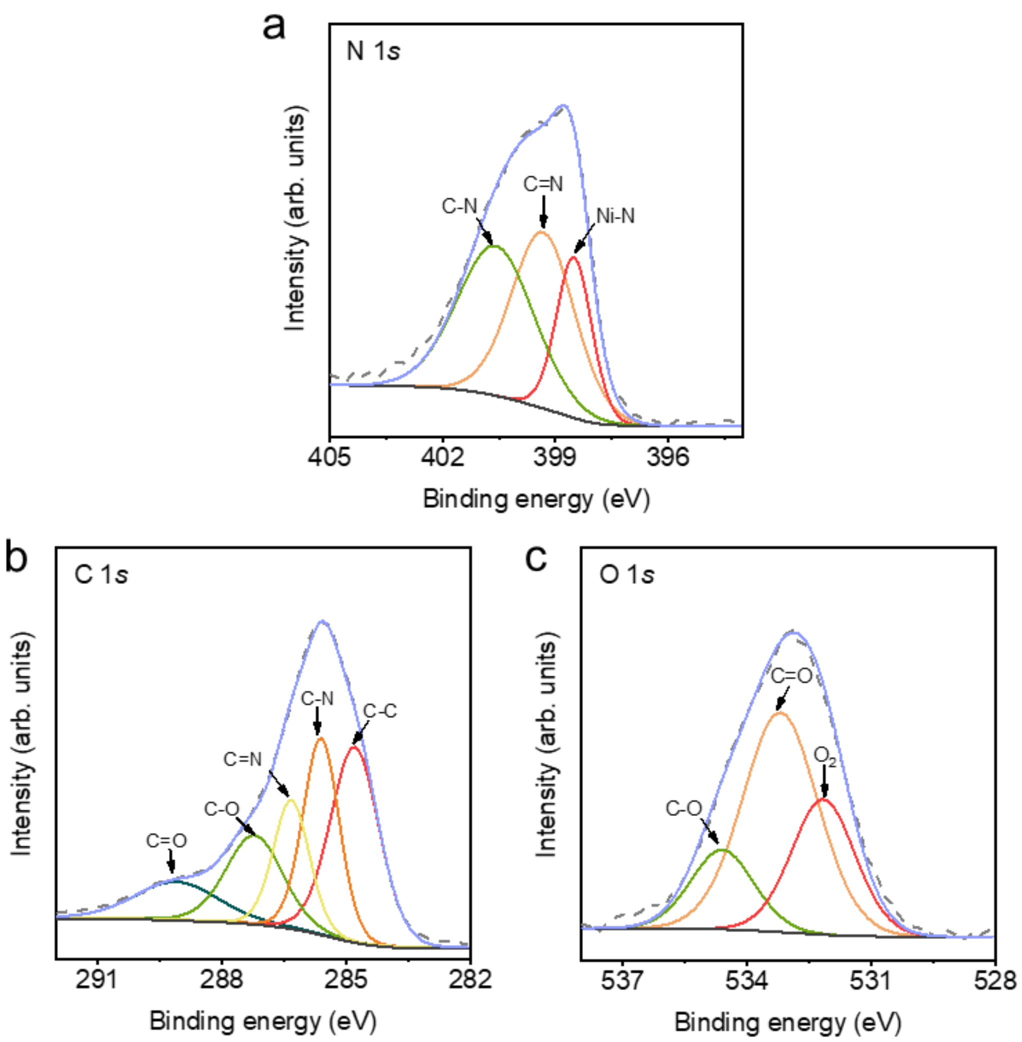

Supplementary Fig. 6. Deconvoluted high-resolution C 1*s*, N 1*s*, and O 1*s* XPS spectra of Ni-DAB. **a**, C 1*s*. **b**, N 1*s*. **c**, O 1*s*. “arb. units” refers to arbitrary units.

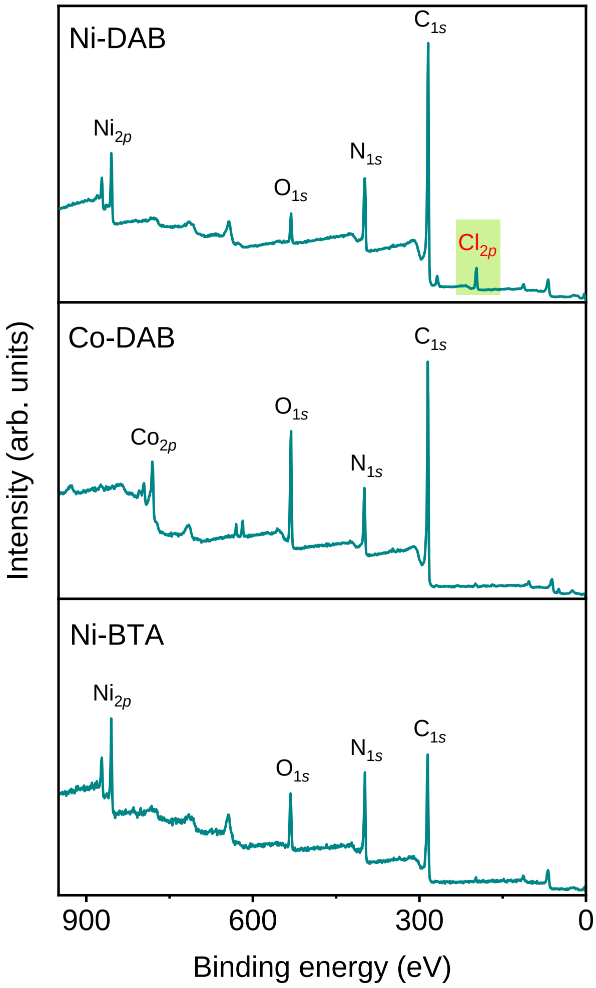

Supplementary Fig. 7. XPS survey of Ni-DAB, Co-DAB and Ni-BTA. Ni-DAB contains charge-balancing anions (Cl^-) and no charge-balancing anions were observed in Co-DAB and Ni-BTA. “arb. units” refers to arbitrary units.

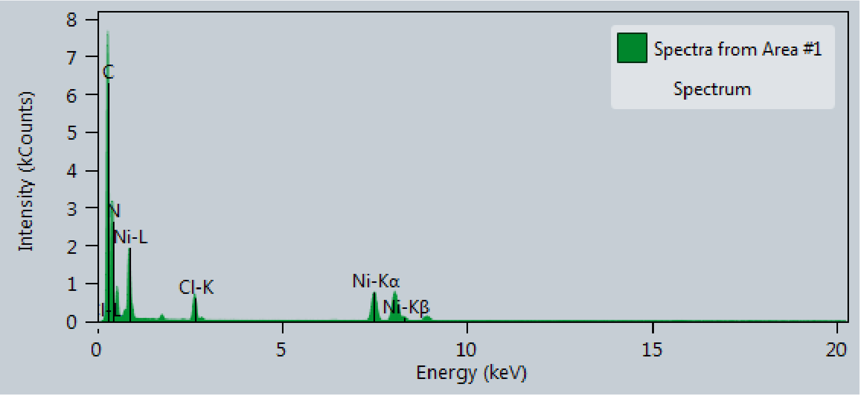

Supplementary Fig. 8. EDS spectrum of Ni-DAB. Except for C, N and Ni, the charge-balancing anions (Cl^-) was also observed.

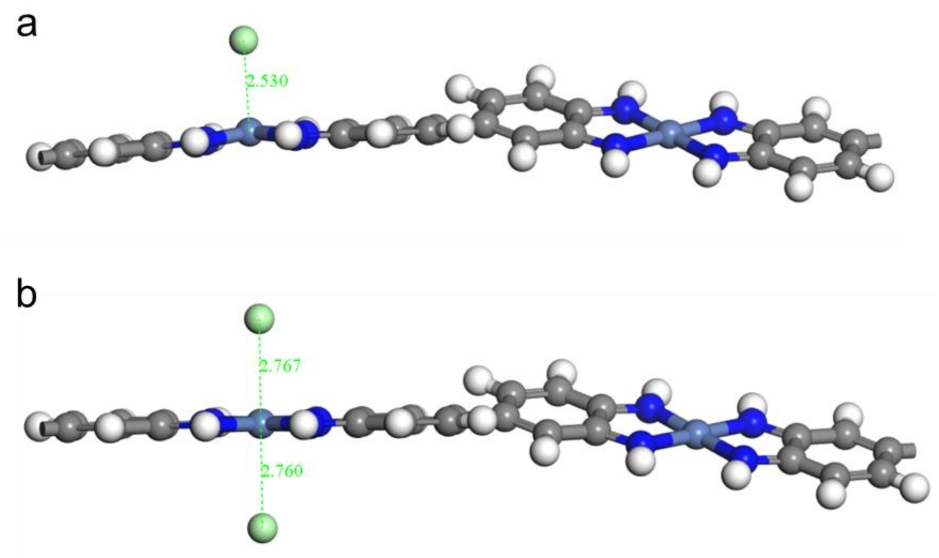

Supplementary Fig. 9. Structures of single (**a**) and two (**b**) Cl ion(s) adsorbed onto the metal center of Ni-DAB in an axial adsorption manner. The distances between Ni and Cl were too far to be coordinated. The white, grey, blue, green, and light blue balls represent H, C, N, Cl, and Ni atoms, respectively.

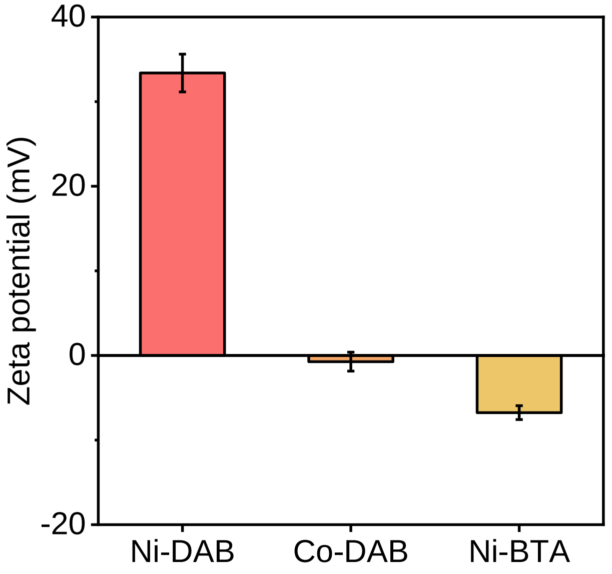

Supplementary Fig. 10. Zeta potential of Ni-DAB, Co-DAB and Ni-BTA (n = 3 independent experiments). All data are presented as mean ± SD.

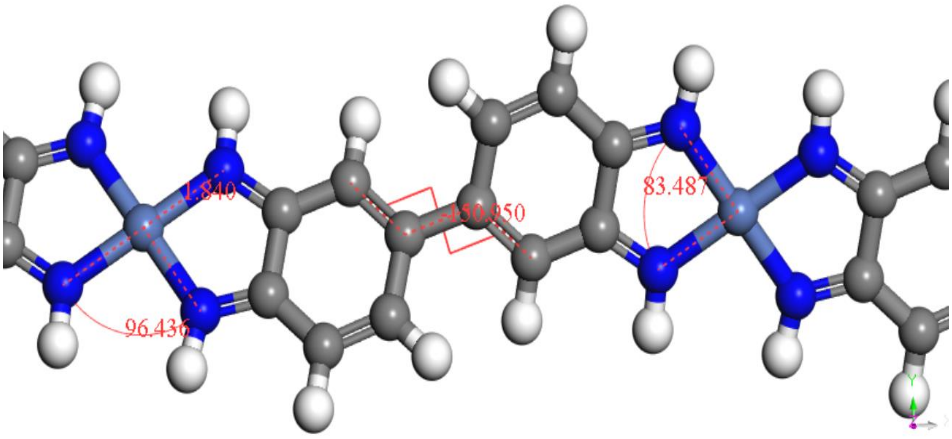

Supplementary Fig. 11. Ni-DAB fitting model, one Ni metal center coordinated with multiple nitrogen atoms (ca. 1.84 Å) in the first shell within one plane.

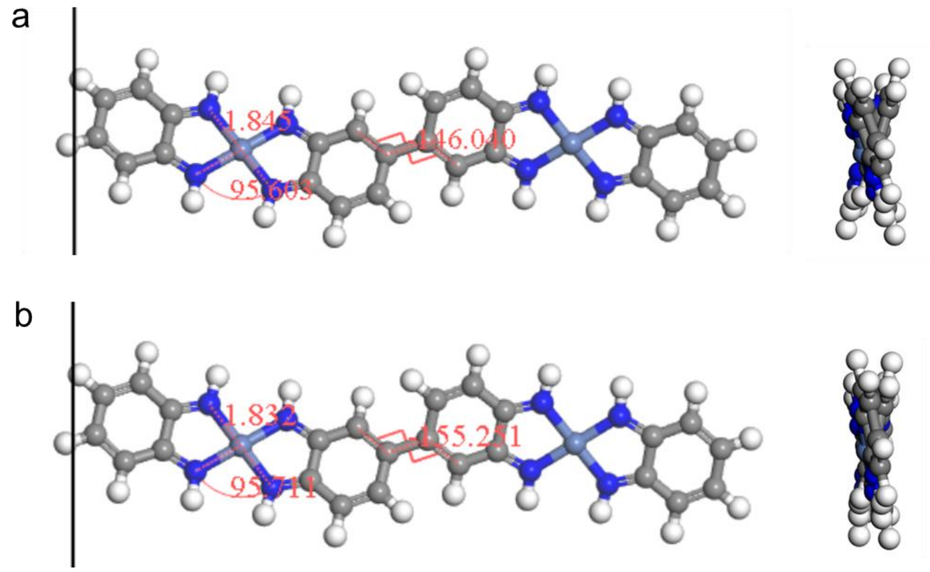

Supplementary Fig. 12. Stable configuration of Ni-DAB imitated by the first principle of molecular dynamics (MD). After 500 steps of MD at 300K, the final frame was extracted and optimized, which showed the same configuration as the initial one. **a**, Before MD. **b**, After MD. The white, grey, blue, and light blue balls represent H, C, N, and Ni atoms, respectively.

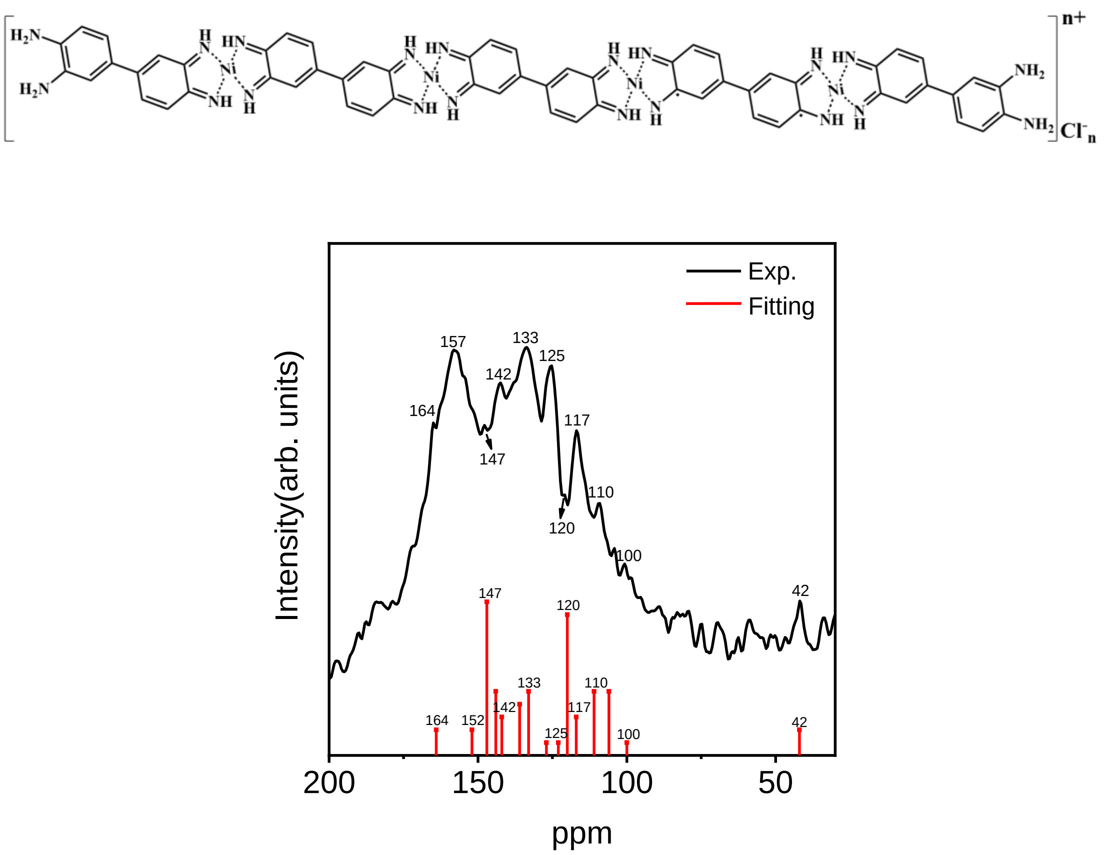

Supplementary Fig. 13. Chain structure of Ni-DAB and Solid ^{13}C NMR spectrum of Ni-DAB and fitting NMR spectrum. “arb. units” refers to arbitrary units.

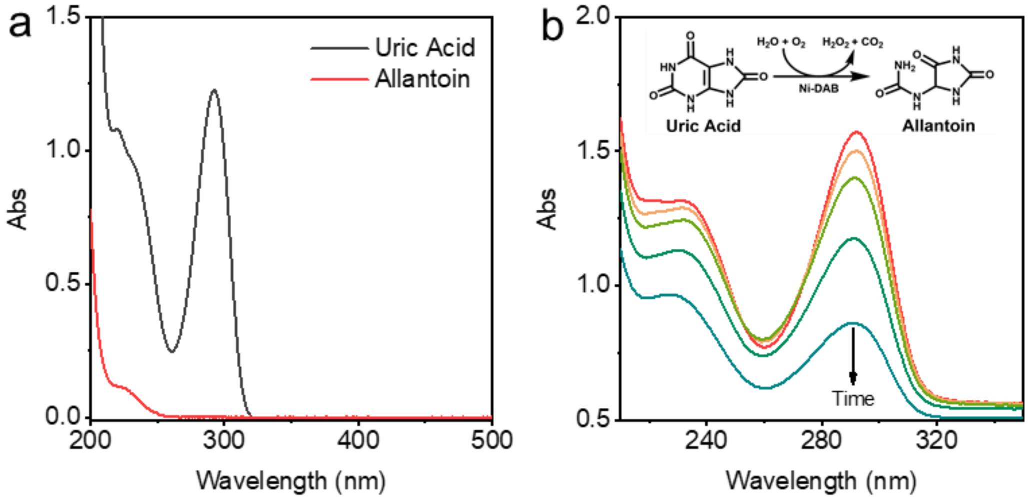

Supplementary Fig. 14. a, UV−vis absorption spectra of uric acid and allantoin. **b,** UV-vis absorption spectra over time in the presence of 0.1 mM UA and 50 μg mL^{-1} Ni-DAB in H_2O.

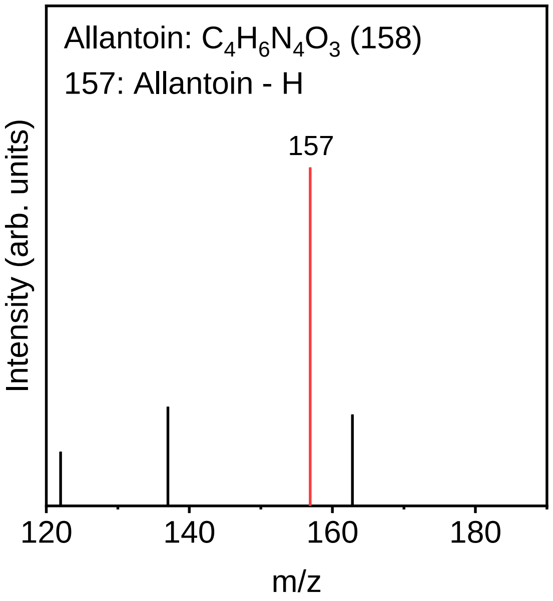

Supplementary Fig. 15. Mass spectra of UA and catalytic product of UA by natural UOX (from arthrobacter protophormiae, 10 U/mg). “arb. units” refers to arbitrary units.

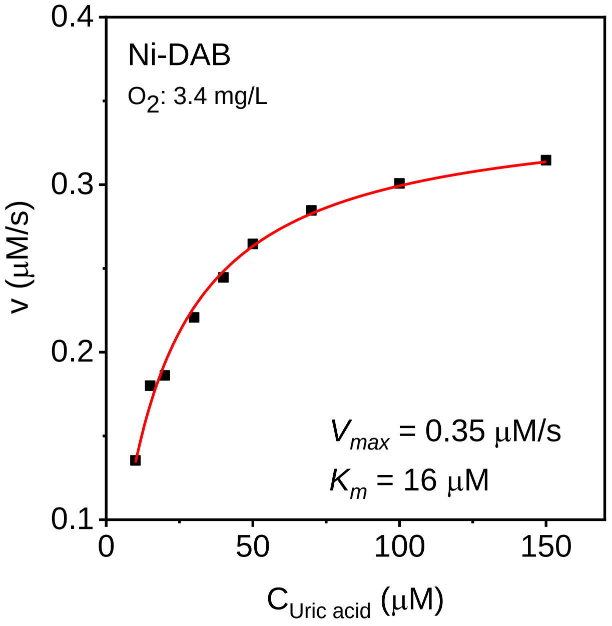

Supplementary Fig. 16. Michaelis–Menten curves for catalytic UA oxidation by Ni-DAB. The solution was bubbled with N_2 for 5 min and the concentration of dissolved O_2 was 3.4 mg/L. Ni-DAB catalyzes UA oxidation with dependence on oxygen concentration like natural oxidase, and the catalytic rate slows down when the oxygen content in the solution decreases.

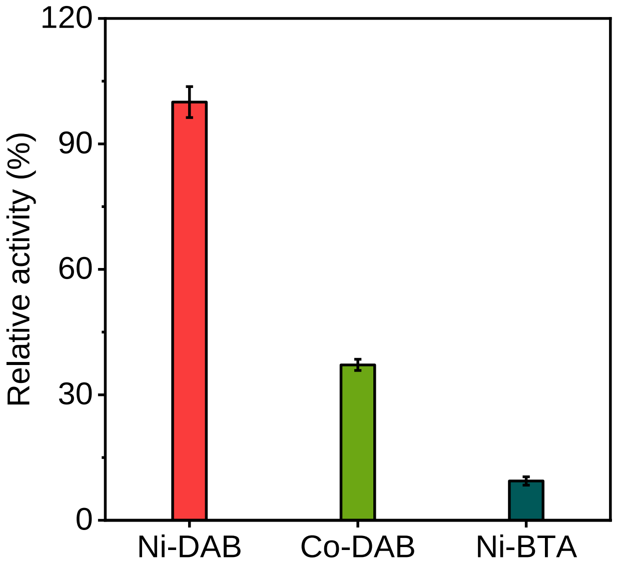

Supplementary Fig. 17. UOX-like activity of various analogous CPs. The UOX-like activity of samples was calculated based on the UV-vis spectroscopy intensity. The concentration of the catalyst was 50 µg mL^{-1} (n = 3 independent experiments). All data are presented as mean ± SD.

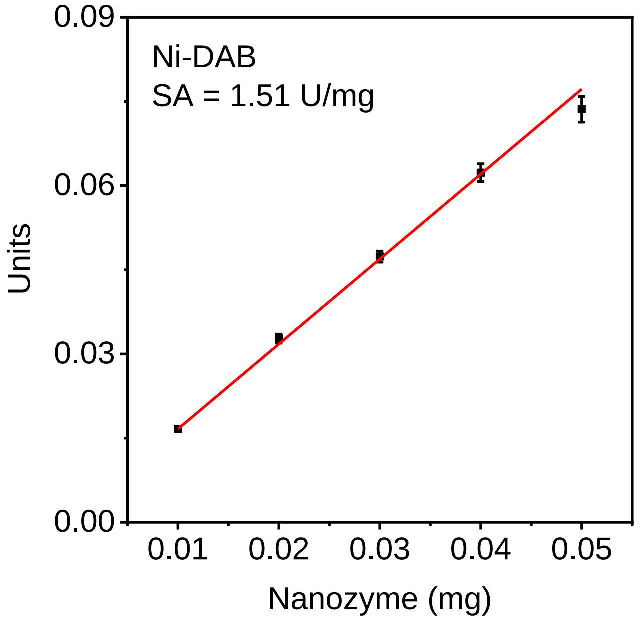

Supplementary Fig. 18. Characterization of the specific activity (SA) of Ni-DAB. The solution was saturated with air (O_2: 8.5 mg/L) (n = 3 independent experiments). All data are presented as mean ± SD.

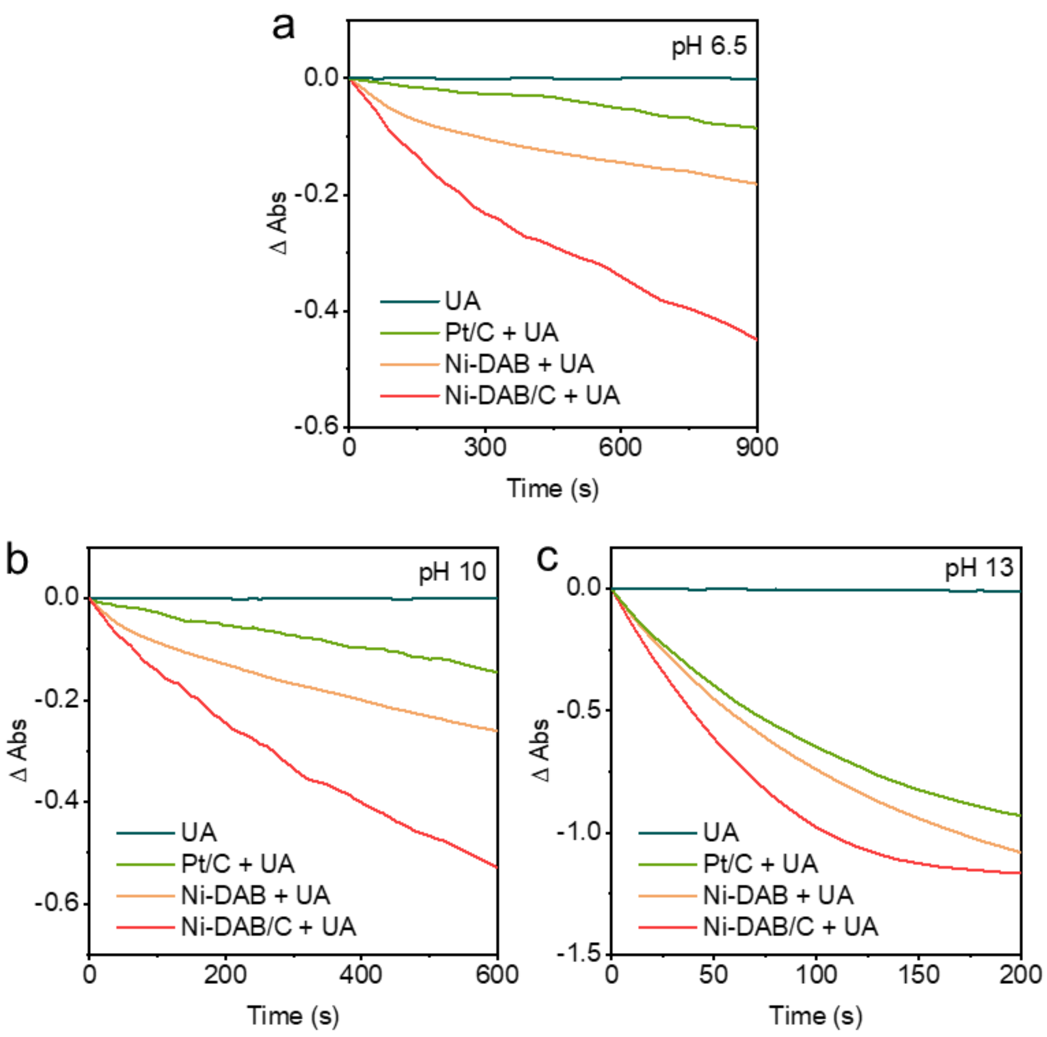

Supplementary Fig. 19. Time-dependent absorbance changes of UA (λ = 293 nm, 0.1 mM) catalyzed by Ni-DAB, Ni-DAB/C and Pt/C with different pH. **a,** pH 6.5. **b,** pH 10. **c,** pH 13. The concentration of the catalyst was 25 μg mL^{-1}.

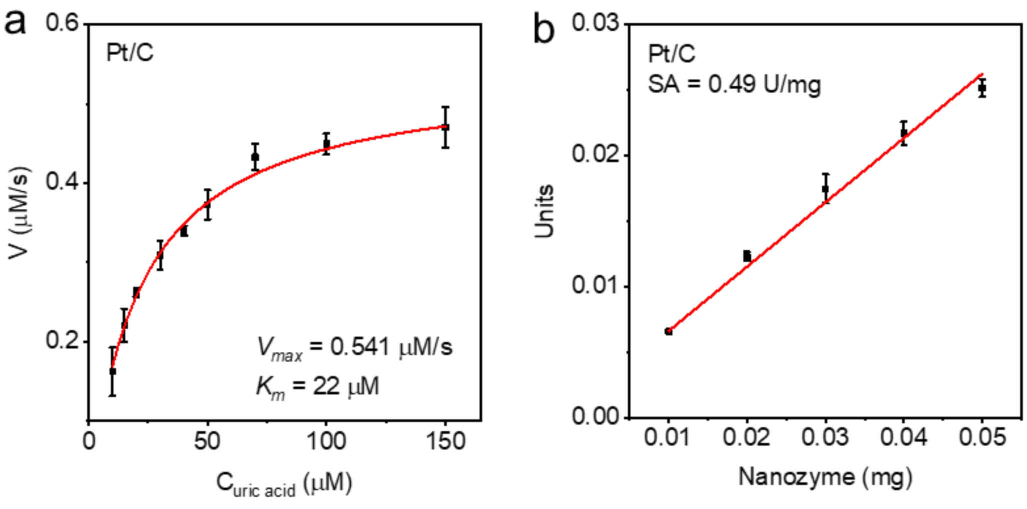

Supplementary Fig. 20. a, Michaelis–Menten curves for UA catalysis by Pt/C. **b,** Characterization of the specific activity of Pt/C. The solution was saturated with air (O_2: 8.5 mg/L) (n = 3 independent experiments). All data are presented as mean ± SD.

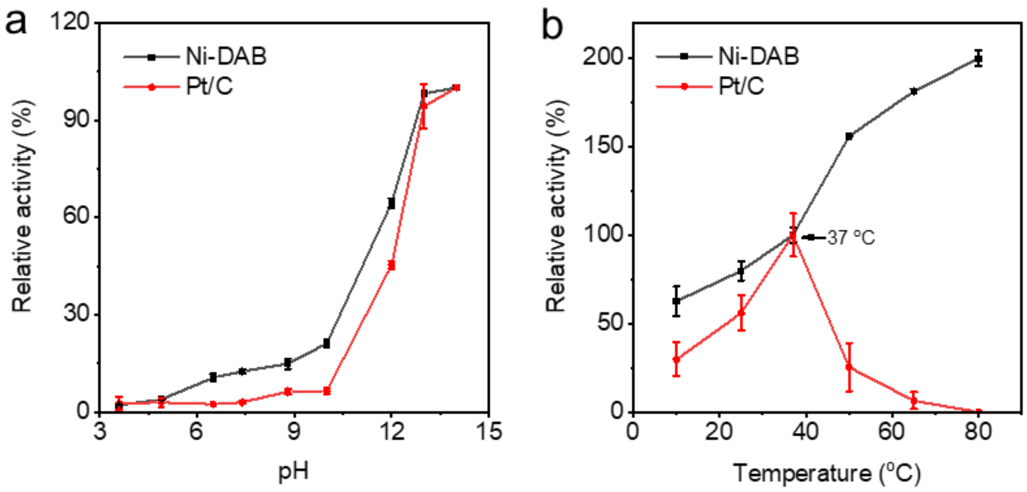

Supplementary Fig. 21. UOX-like activities of Ni-DAB at different pH values **a**, and temperatures **b**. (n = 3 independent experiments). All data are presented as mean ± SD.

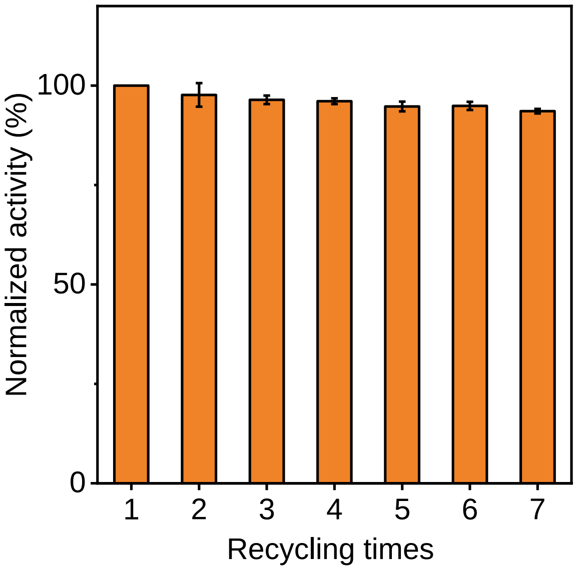

Supplementary Fig. 22. Recovery and recyclability of Ni-DAB in the catalytic oxidation of UA (n = 3 independent experiments). All data are presented as mean ± SD.

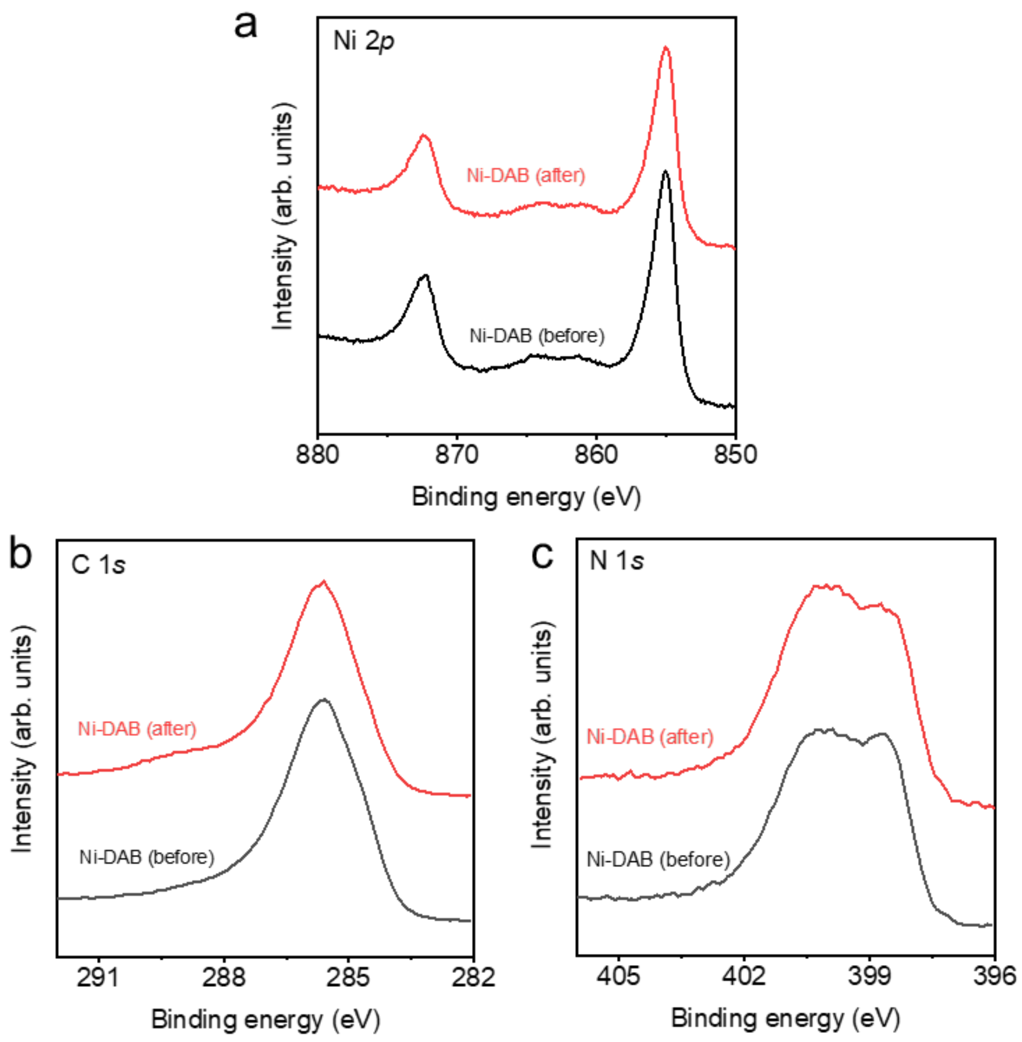

Supplementary Fig. 23. High-resolution XPS spectra of Ni-DAB before and after repetitive catalytic oxidation of UA. **a**, Ni 2*p*. **b**, C 1*s*. **c**, N 1*s*. “arb. units” refers to arbitrary units.

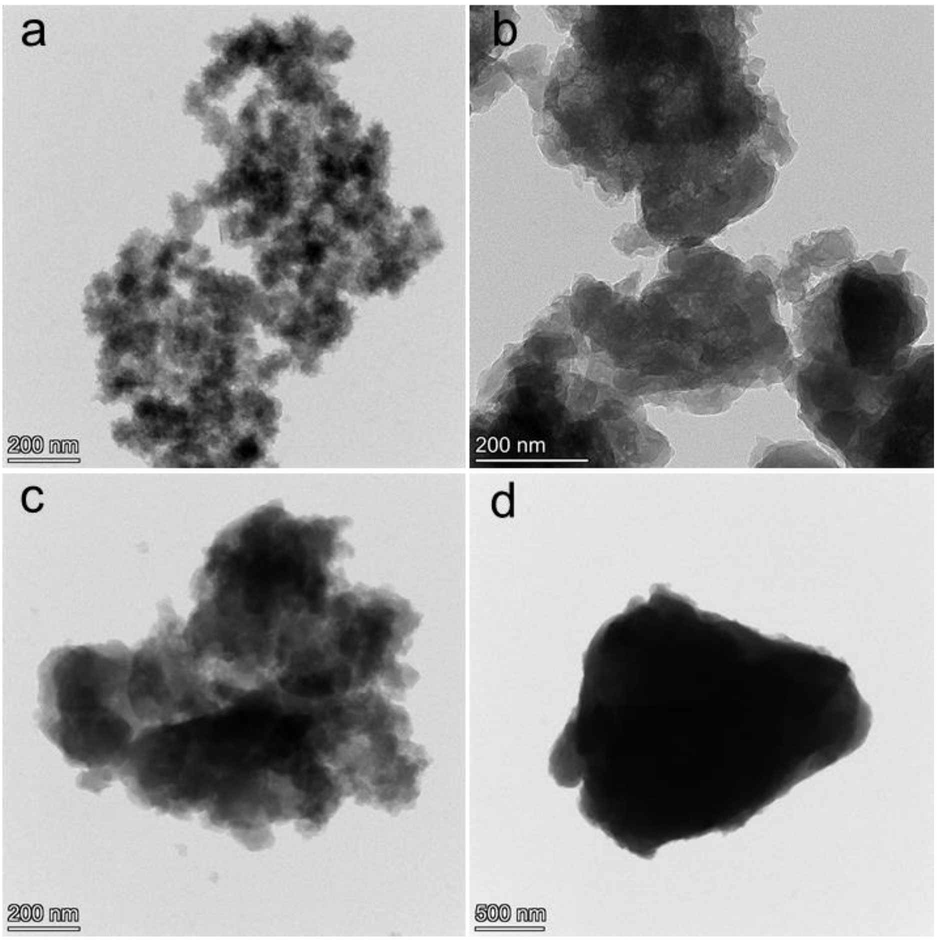

Supplementary Fig. 24. TEM images of Ni-DAB prepared with different amounts of ammonia. **a**, Ni-DAB(1). **b**, Ni-DAB(3). **c**, Ni-DAB(7). **d**, Ni-DAB(11). The number in the brackets was the volume of ammonia (wt. 25%) that was used in preparation of Ni-DAB (See the experimental for other conditions). Experiment was repeated 3 times independently with similar results.

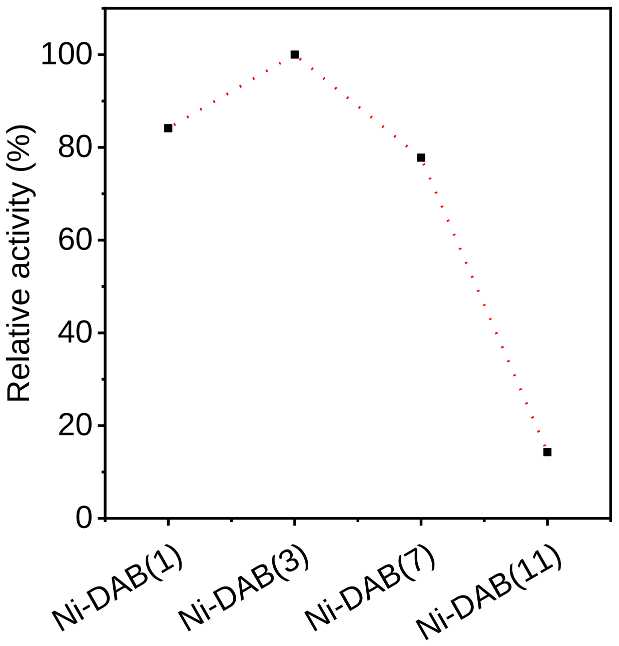

Supplementary Fig. 25. UOX-like activity of Ni-DAB(*x*) (*x* = 1, 3, 7, and 11). *x* was the volume of ammonia (wt. 25%) that was used in preparation of Ni-DAB (See the experimental for other conditions). The UOX-like activity of samples was calculated based on the UV-vis spectroscopy intensity.

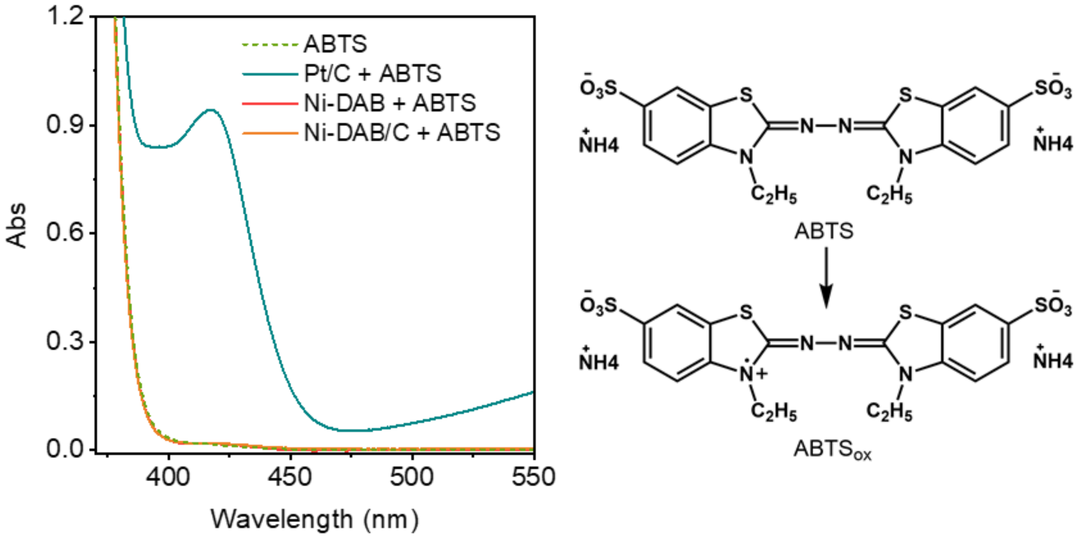

Supplementary Fig. 26. UV−vis absorption spectra of the solutions containing 2,2'-azinobis-(3-ethylbenzthiazoline-6-sulphonate) (ABTS, 0.5 mM) and different catalysts (50 μg mL^{-1}) in NaAc/HAc buffer solution (50 mM, pH 3.6).

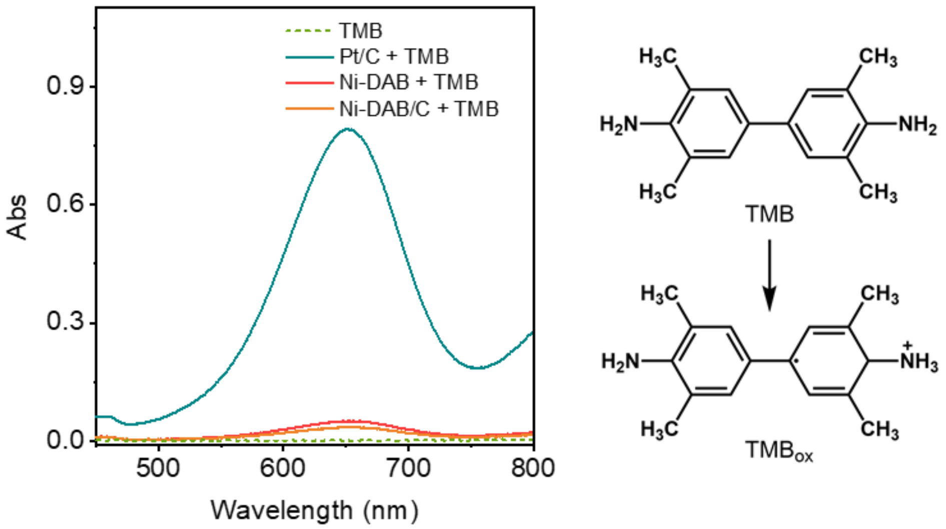

Supplementary Fig. 27. UV−vis absorption spectra of the solutions containing 3,3',5,5'-tetramethylbenzidine (TMB, 0.5 mM) and different catalysts (50 μg mL^{-1}) in NaAc/HAc buffer solution (50 mM, pH 5).

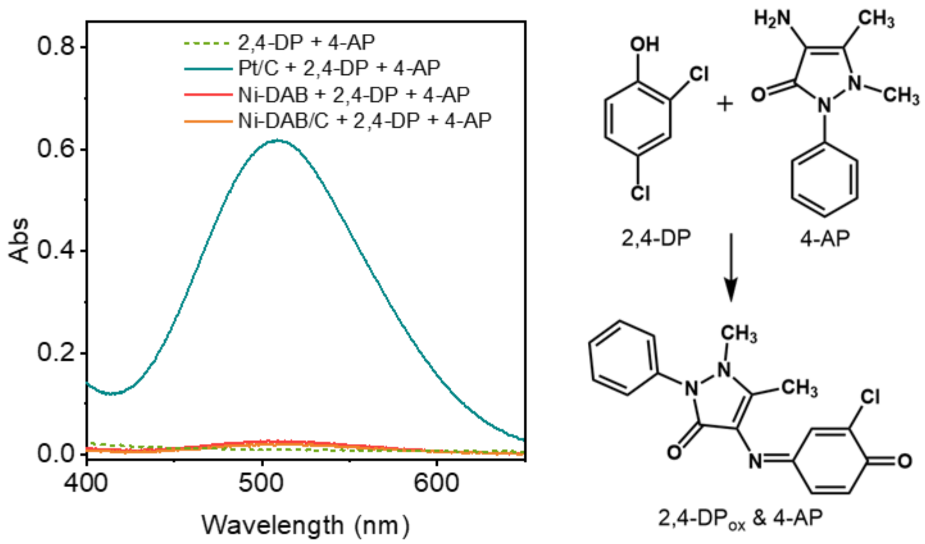

Supplementary Fig. 28. UV−vis absorption spectra of the solutions containing 2,4-dichlorophenol (2,4-DP, 0.5 mM) and different catalysts (50 μg mL^{-1}) in HEPES-NaOH buffer solution (50 mM, pH 7.4). The concentration of chromogenic agent-4-aminoantipyrine (4-AP) was 0.5 mM.

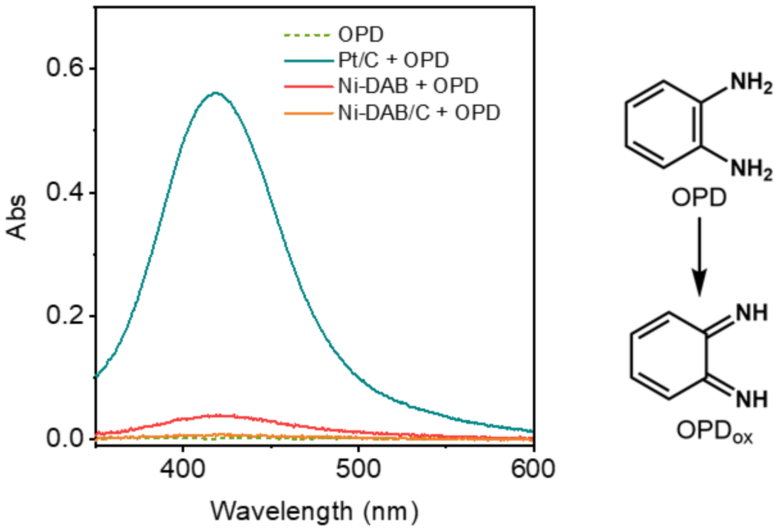

Supplementary Fig. 29. UV−vis absorption spectra of the solutions containing o-phenylenediamine (OPD, 0.5 mM) and different catalysts (50 μg mL^{-1}) in HEPES-NaOH buffer solution (50 mM, pH 7.4).

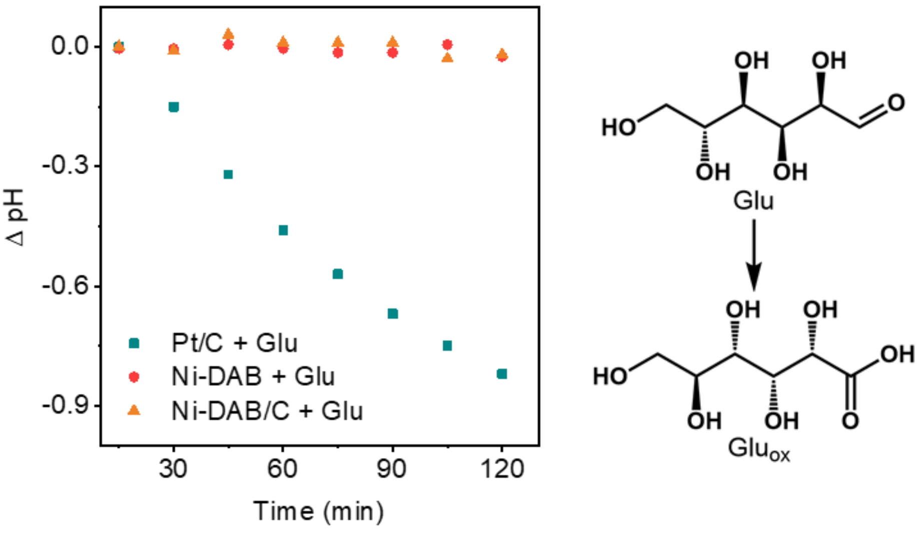

Supplementary Fig. 30. pH changes of glucose (Glu) solution (0.1 M) catalyzed by Ni-DAB, Ni-DAB/C and Pt/C (50 μg mL^{-1}).

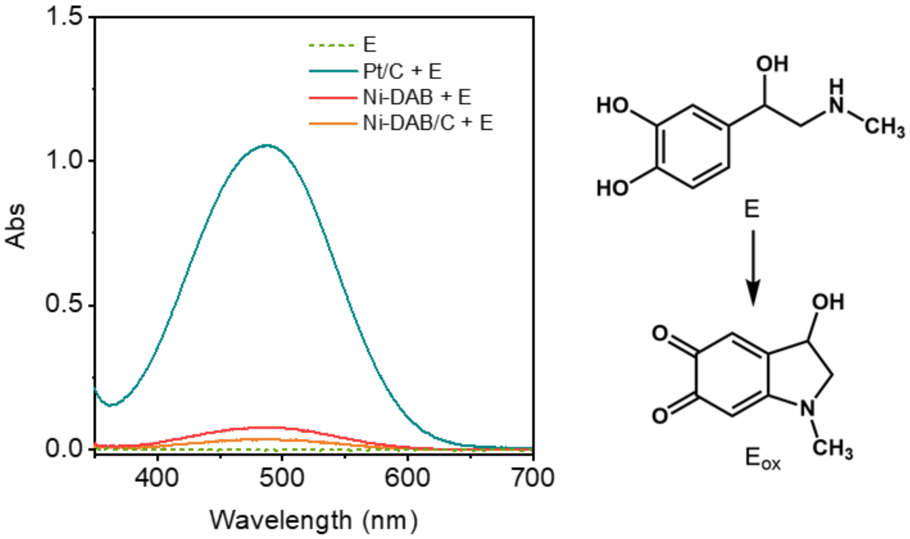

Supplementary Fig. 31. UV−vis absorption spectra of the solutions containing epinephrine (E, 0.5 mM) and different catalysts (50 μg mL^{-1}) in HEPES-NaOH buffer solution (50 mM, pH 7.4).

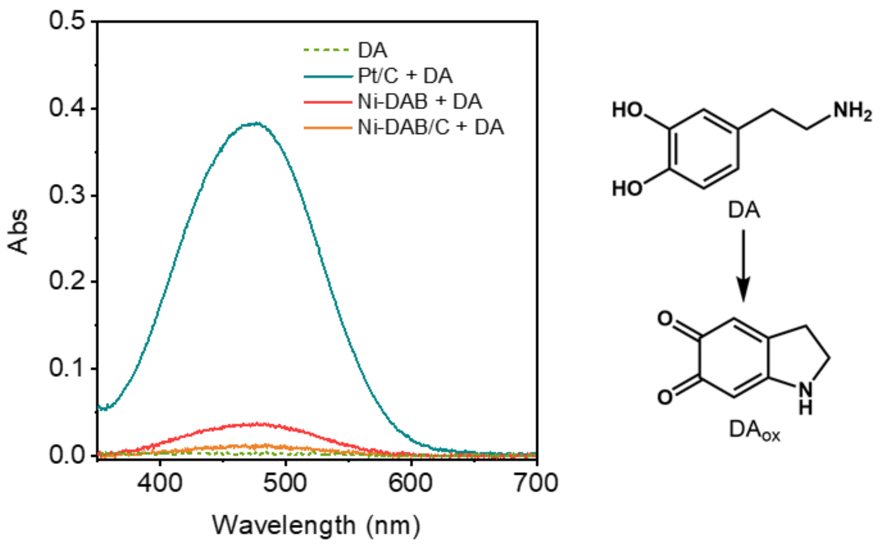

Supplementary Fig. 32. UV−vis absorption spectra of the solutions containing dopamine (DA, 0.5 mM) and different catalysts (50 μg mL^{-1}) in HEPES-NaOH buffer solution (50 mM, pH 6.5).

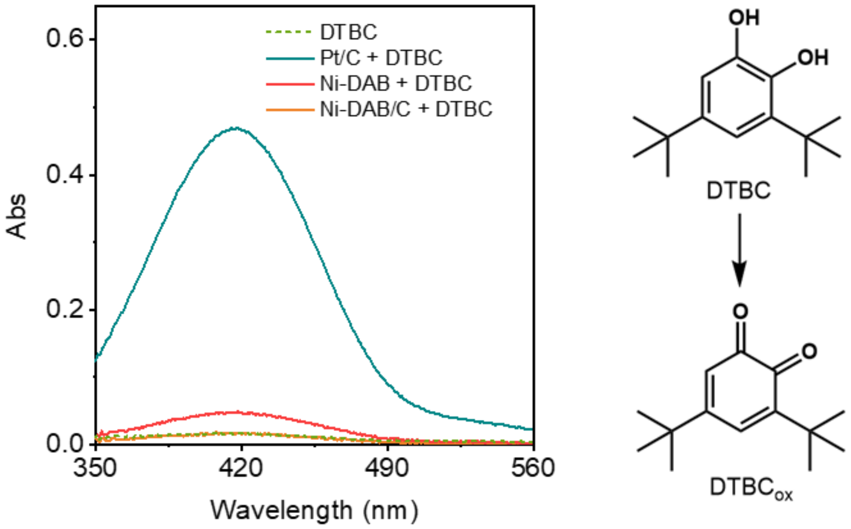

Supplementary Fig. 33. UV−vis absorption spectra of the solutions containing 3,5-ditert-butyl catechol (DTBC, 0.5 mM) and different catalysts (50 μg mL^{-1}) in HEPES-NaOH buffer solution (50 mM, pH 7.4).

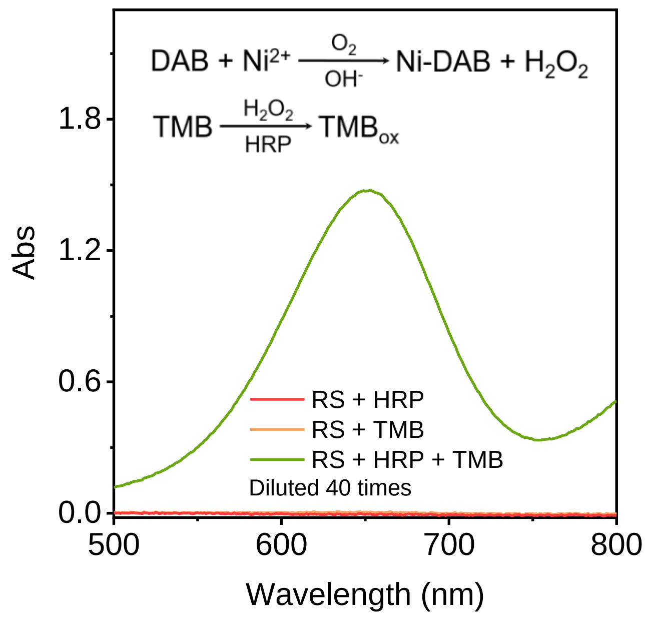

Supplementary Fig. 34. UV-vis absorption spectra of solutions with combinations of TMB, HRP, and the supernatant reaction solution (RS, diluted 40 times) for the preparation of Ni-DAB. Inset: diagram of the by-product H_2O_2 produced during the synthesis of Ni-DAB.

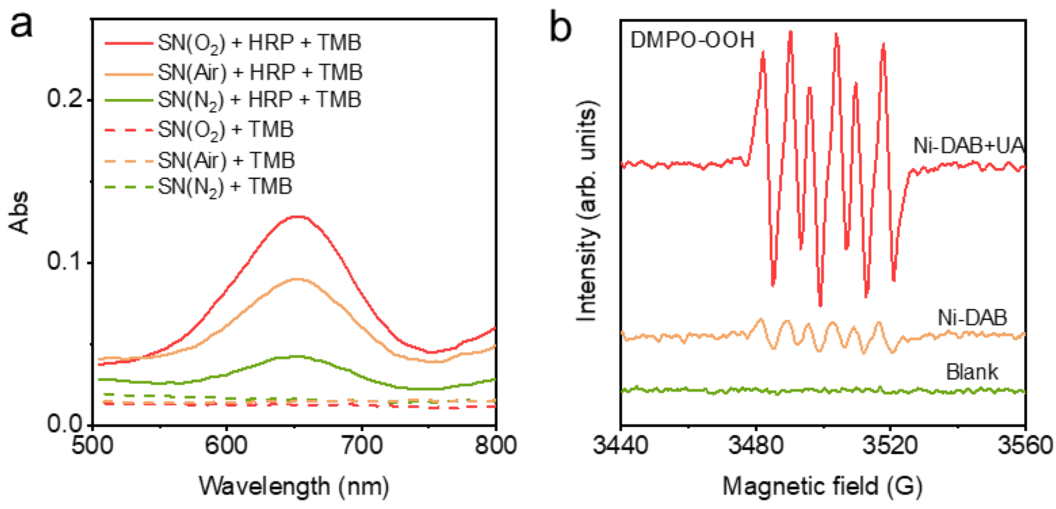

Supplementary Fig. 35. a, UV-vis absorption spectra of combinations of Ni-DAB aqueous dispersion supernatant (SN, 50 μg mL^{-1}), HRP, and TMB in O_2-saturated, Air-saturated, and N_2-saturated solutions. **b,** EPR spectra of Ni-DAB (0.5 mg mL^{-1}) and Ni-DAB + UA (1 mM) for $\cdot O_2^-$ in DMPO spin trap aqueous solution. “arb. units” refers to arbitrary units.

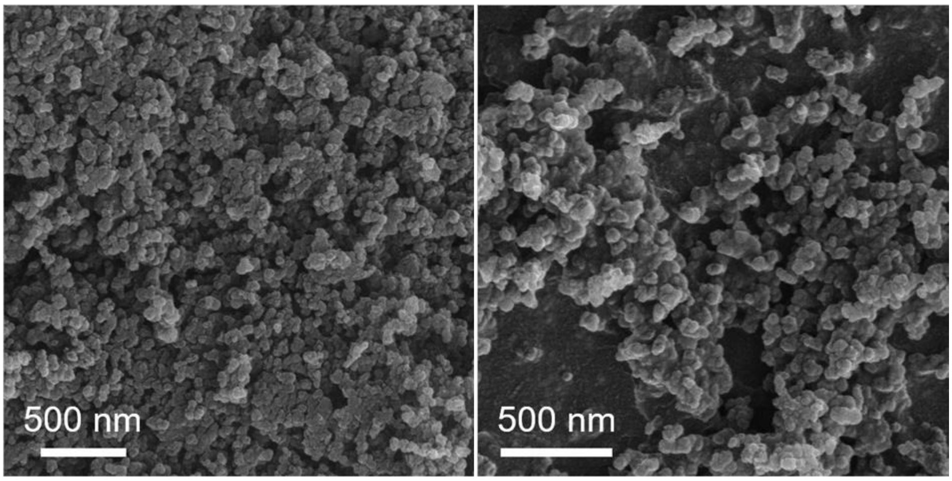

Supplementary Fig. 36. SEM images of Ni-DAB/C. Experiment was repeated 3 times independently with similar results.

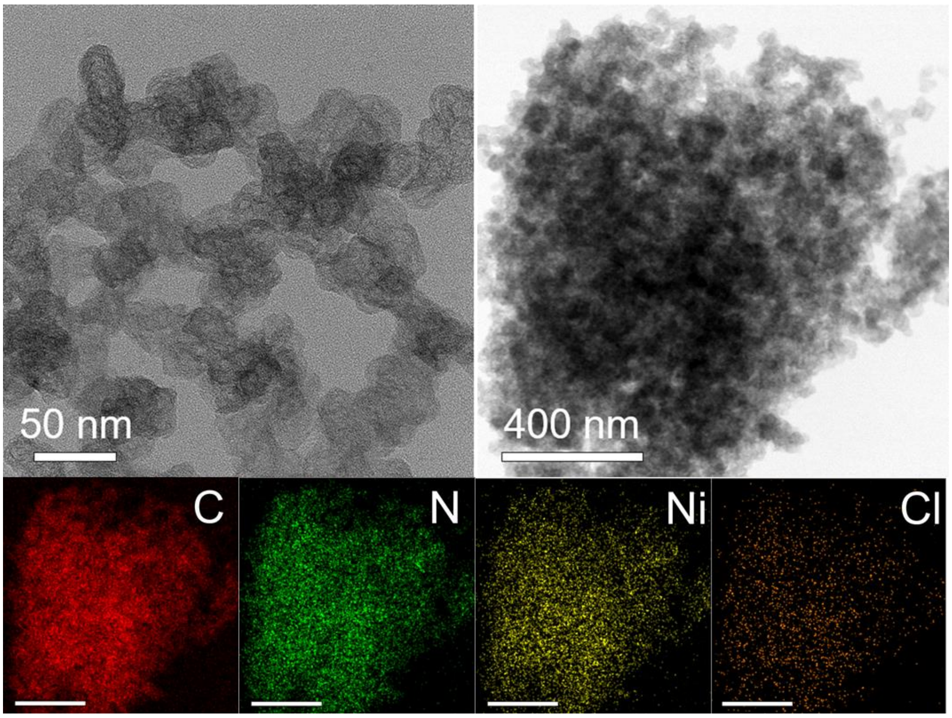

Supplementary Fig. 37. TEM images of Ni-DAB/C and corresponding TEM-EDS elemental mapping images, showing the uniformly distributed C, N, Ni, and Cl elements in Ni-DAB/C. Scale bars: 400 nm. Experiment was repeated 3 times independently with similar results.

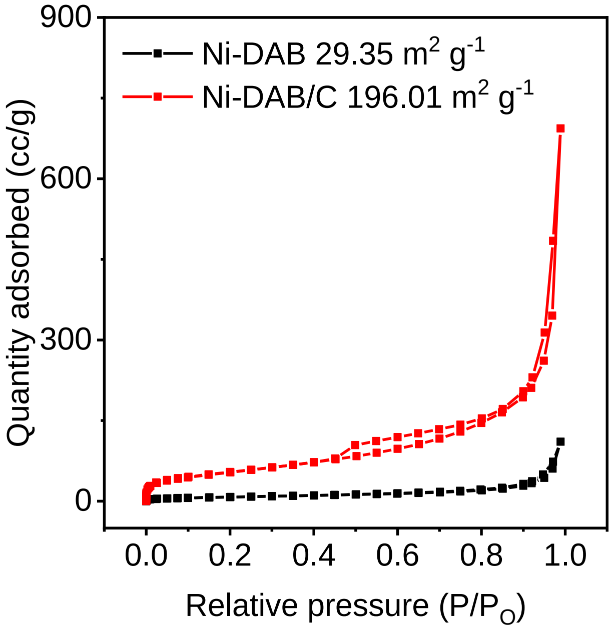

Supplementary Fig. 38. Nitrogen adsorption−desorption isotherms at 77 K of Ni-DAB and Ni-DAB/C. Before starting the adsorption measurements, the sample was degassed at 150 ºC for 12 h.

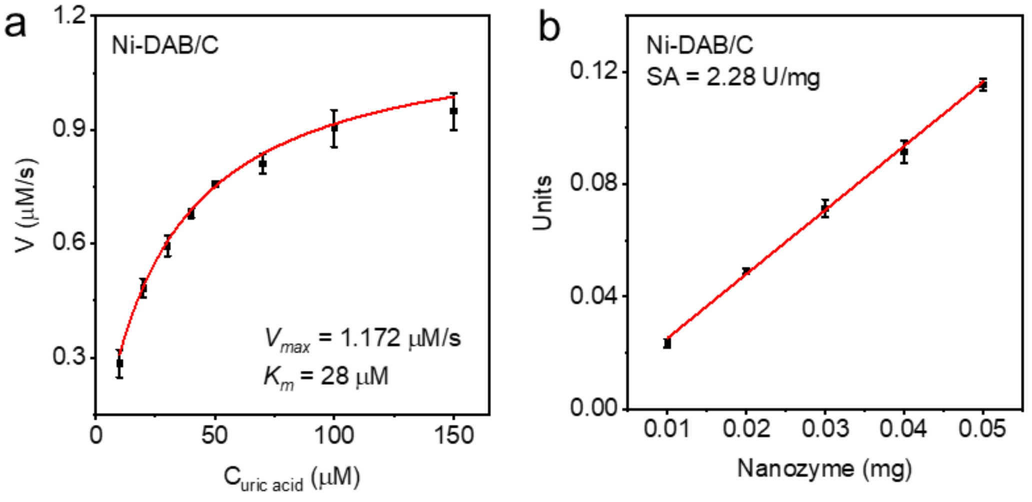

Supplementary Fig. 39. a, Michaelis–Menten curves for UA catalysis by Ni-DAB/C. **b,** Characterization of the specific activity of Ni-DAB/C (n = 3 independent experiments). All data are presented as mean ± SD.

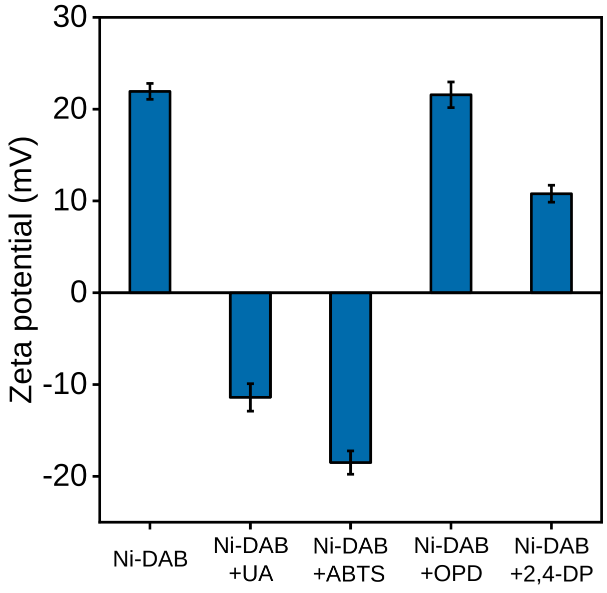

Supplementary Fig. 40. Zeta potential changes of Ni-DAB with different substrates added (n = 3 independent experiments). All data are presented as mean ± SD.

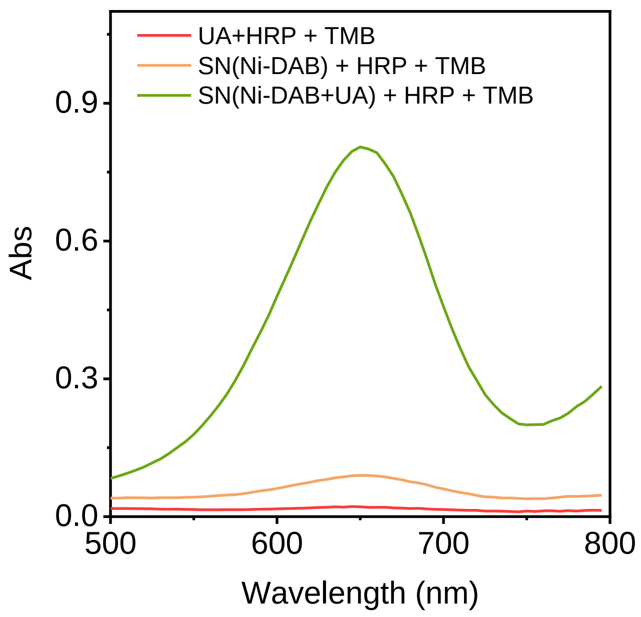

Supplementary Fig. 41. UV-vis absorption spectra of solutions with combinations of TMB, HRP, and UA reaction products solution catalyzed by Ni-DAB.

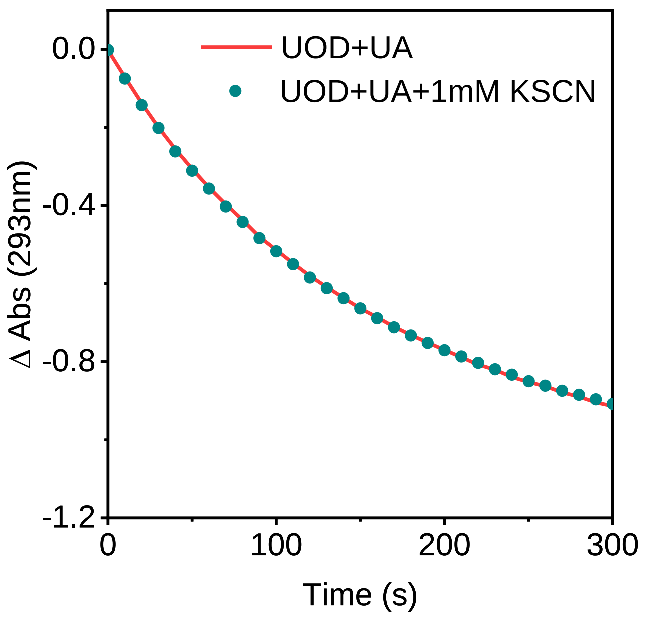

Supplementary Fig. 42. Time-dependent absorbance changes of UA (λ = 293 nm, 0.1 mM) catalyzed by natural UOX before and after SCN^{-} poisoning.

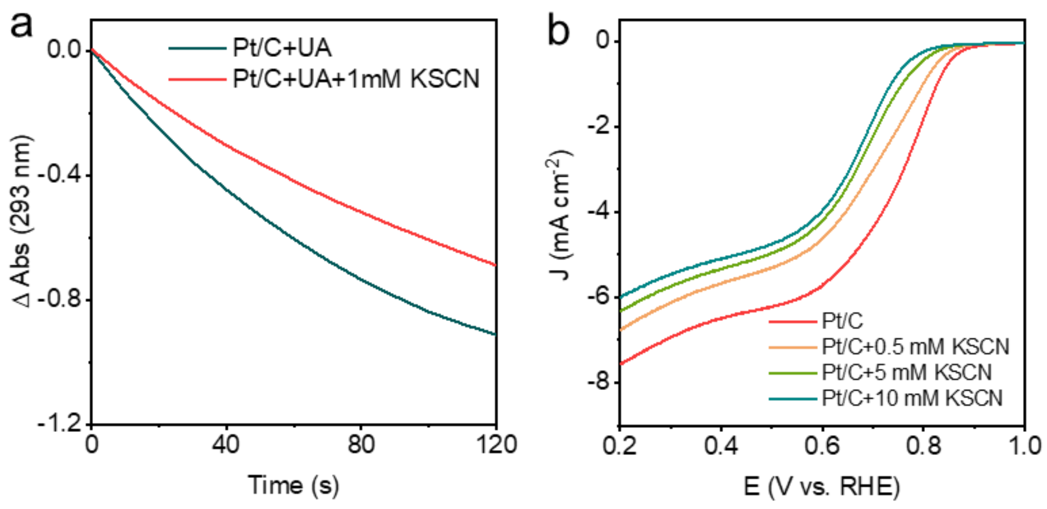

Supplementary Fig. 43. a, Time-dependent absorbance changes of UA (λ = 293 nm, 0.1 mM) catalyzed by Pt/C before and after SCN^- poisoning. The concentration of Pt/C was 50 μg mL^{-1}. **b,** ORR activity of Pt/C before and after SCN^- poisoning examined by LSV curves using RDE in 0.1 M KOH.

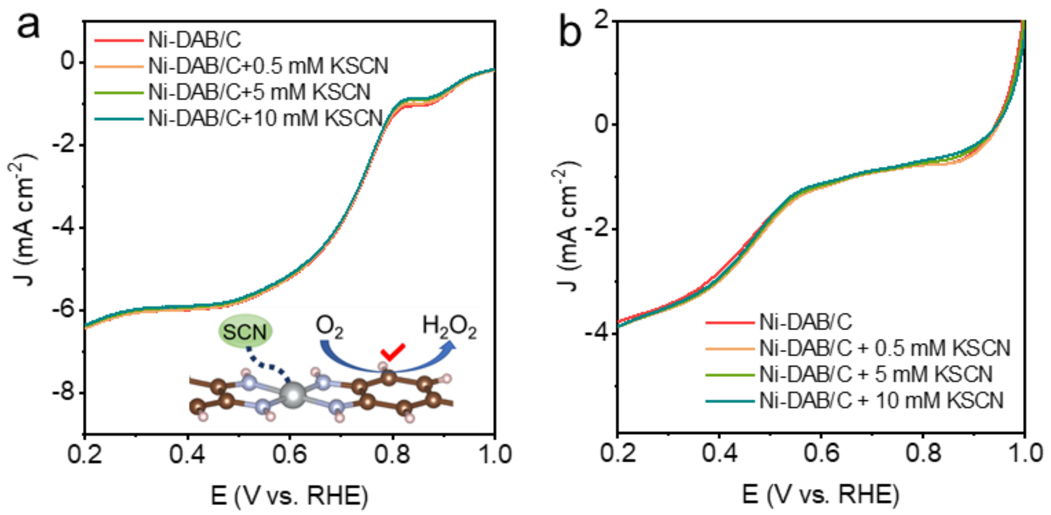

Supplementary Fig. 44. ORR activity of Ni-DAB/C before and after SCN^- poisoning examined by LSV curves using RDE. **a,** in alkaline 0.1 M KOH. **b,** in neutral 0.05 M PBS.

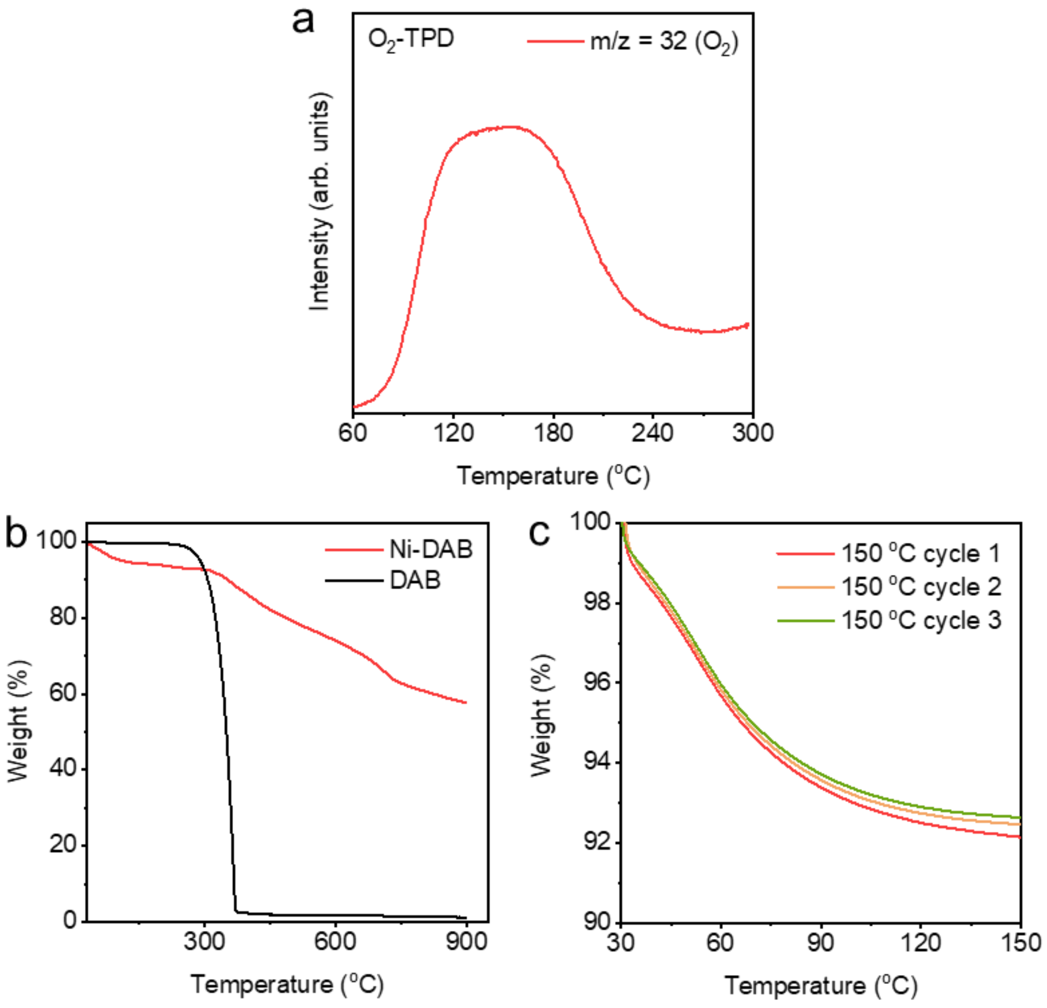

Supplementary Fig. 45. a, O_2-TPD-MS profiles of Ni-DAB. **b,** TGA curves of Ni-DAB and DAB. **c,** TGA cycle curves of Ni-DAB. "arb. units" refers to arbitrary units.

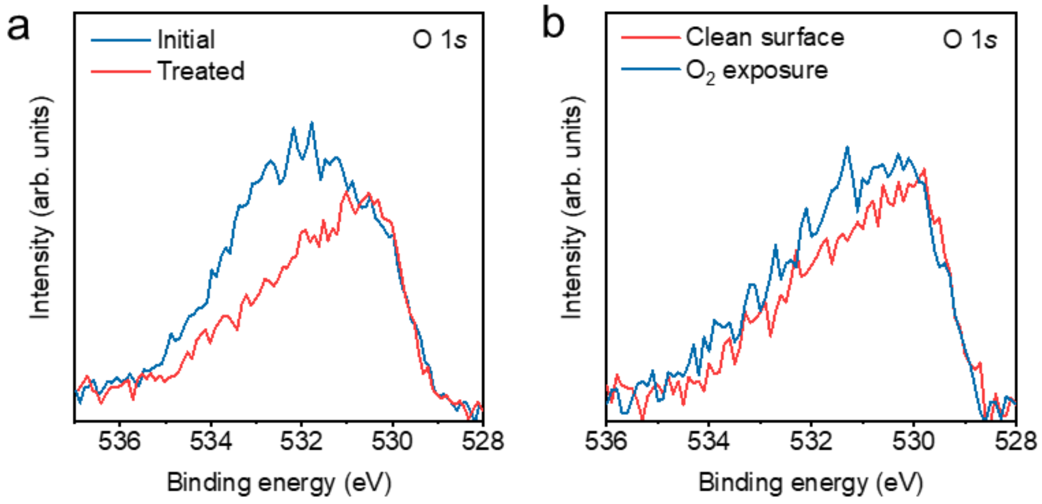

Supplementary Fig. 46. a, XPS spectra of O 1*s* before and after thermally treatment at 150 °C in vacuum of Ni-DAB. **b,** XPS spectra of O 1*s* before and after 0.5 mbar O_2 exposure for 30 min of Ni-DAB. “arb. units” refers to arbitrary units.

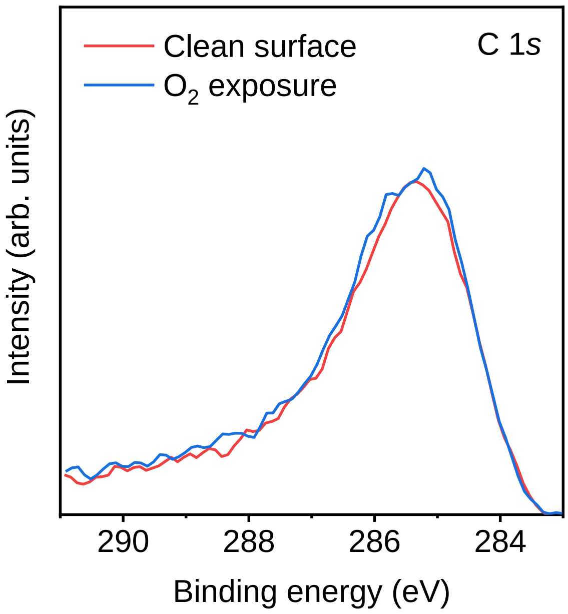

Supplementary Fig. 47. XPS spectra of C 1*s* before and after 0.5 mbar O_2 exposure for 30 min of Ni-DAB. “arb. units” refers to arbitrary units.

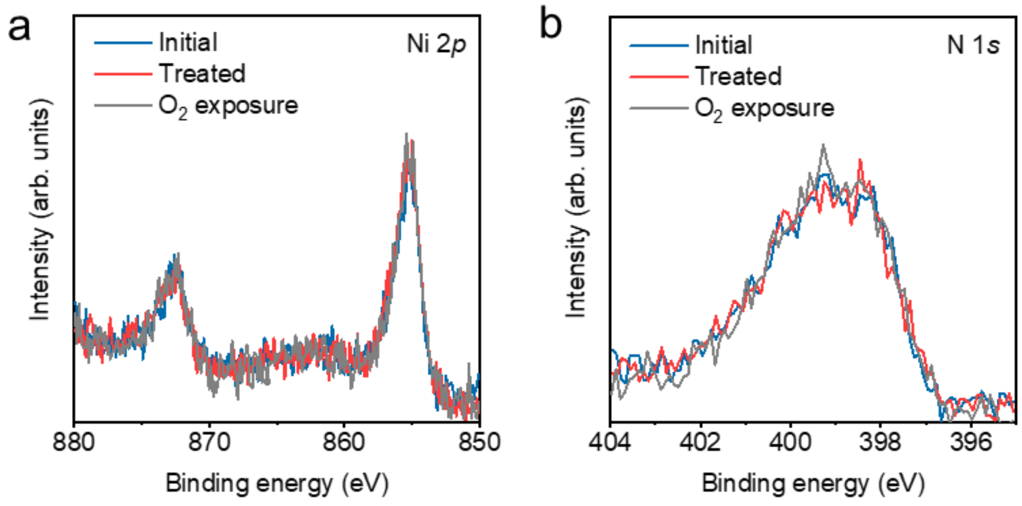

Supplementary Fig. 48. XPS spectra of Ni-DAB at all stages of testing. **a,** Ni 2*p*. **b,** N 1*s*. "arb. units" refers to arbitrary units.

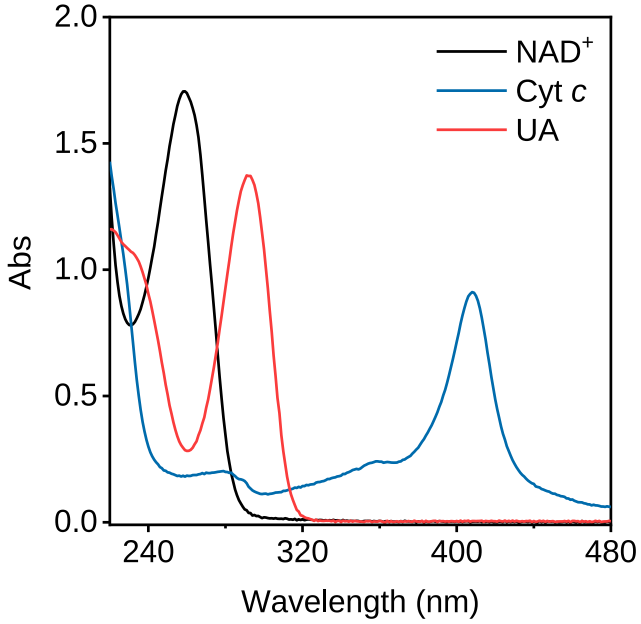

Supplementary Fig. 49. UV−vis absorption spectra of NAD^+, Cyt *c*, and UA.

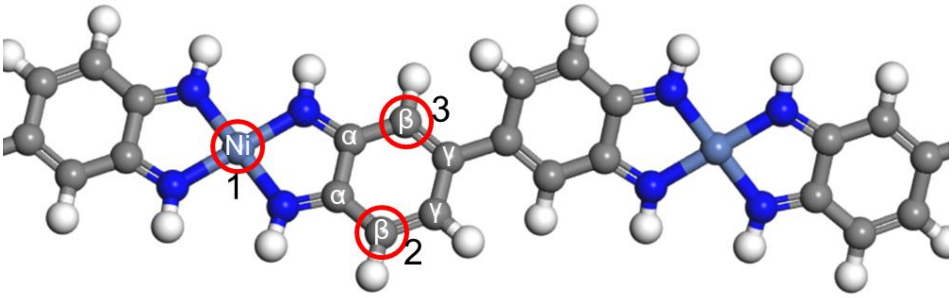

Supplementary Fig. 50. Diagram of possible ORR active sites of Ni-DAB, including Ni metal center (1), beta-C (2), and beta-C (3)

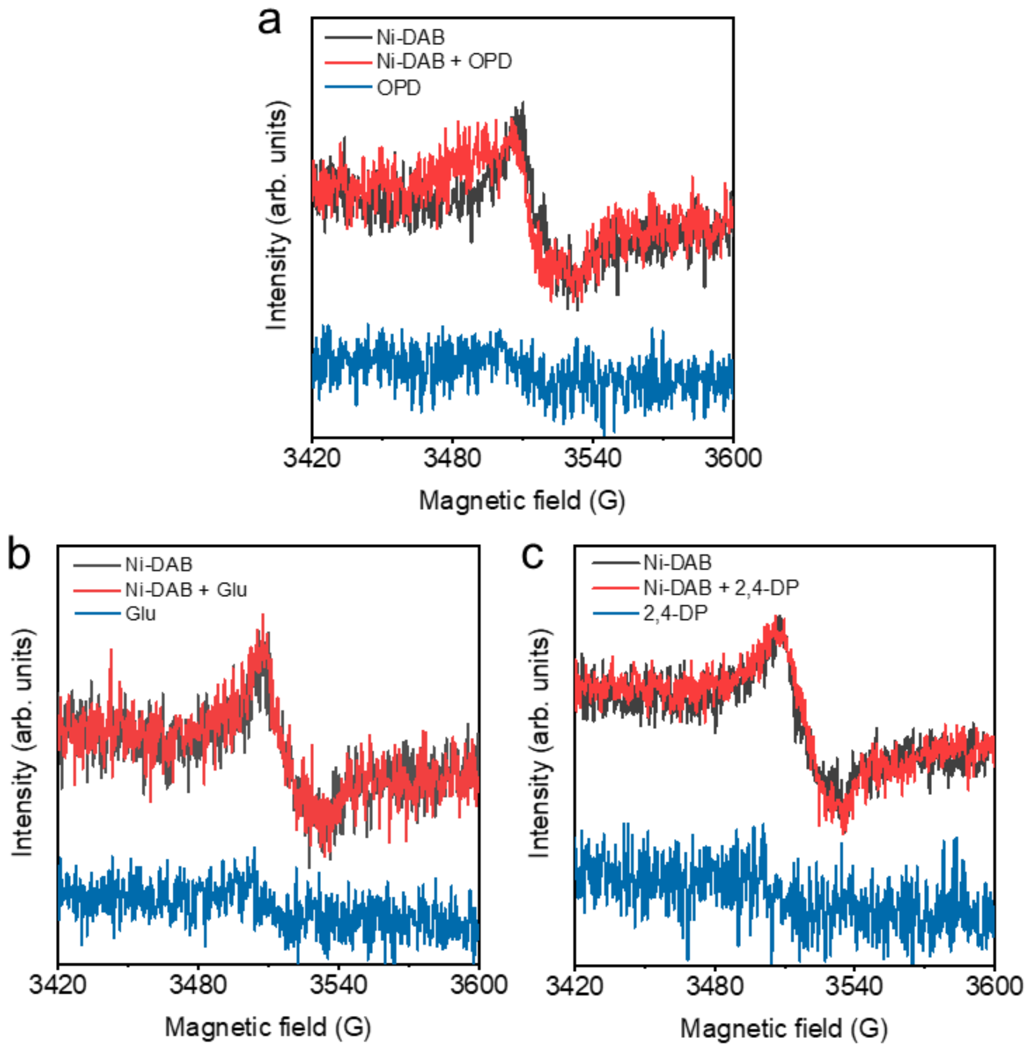

Supplementary Fig. 51. EPR spectra of Ni-DAB with different substrates added under deoxygenation conditions. **a,** OPD. **b,** Glu. **c,** 2,4-DP. “arb. units” refers to arbitrary units.

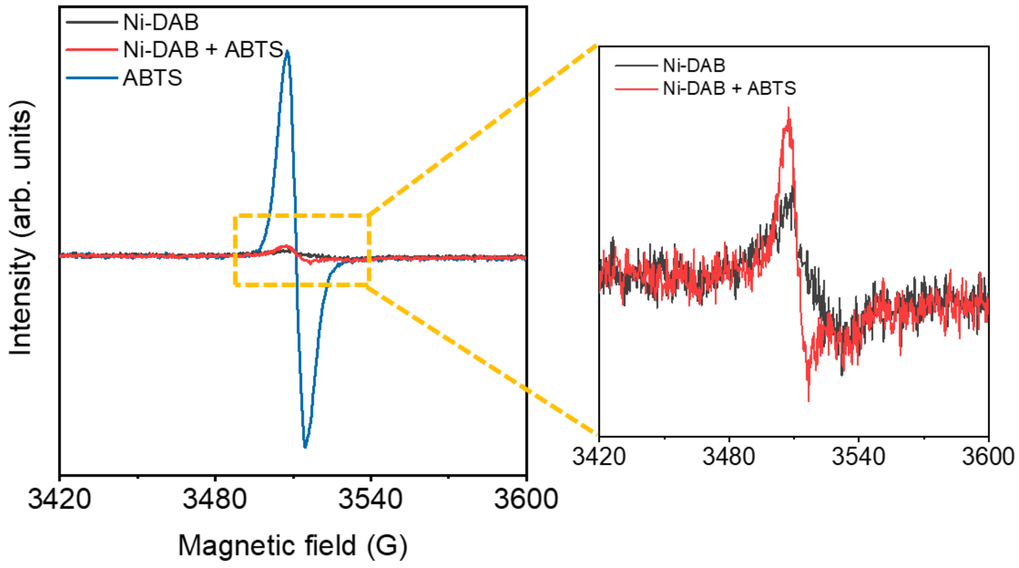

Supplementary Fig. 52. EPR spectra of Ni-DAB with ABTS added under deoxygenation conditions. “arb. units” refers to arbitrary units.

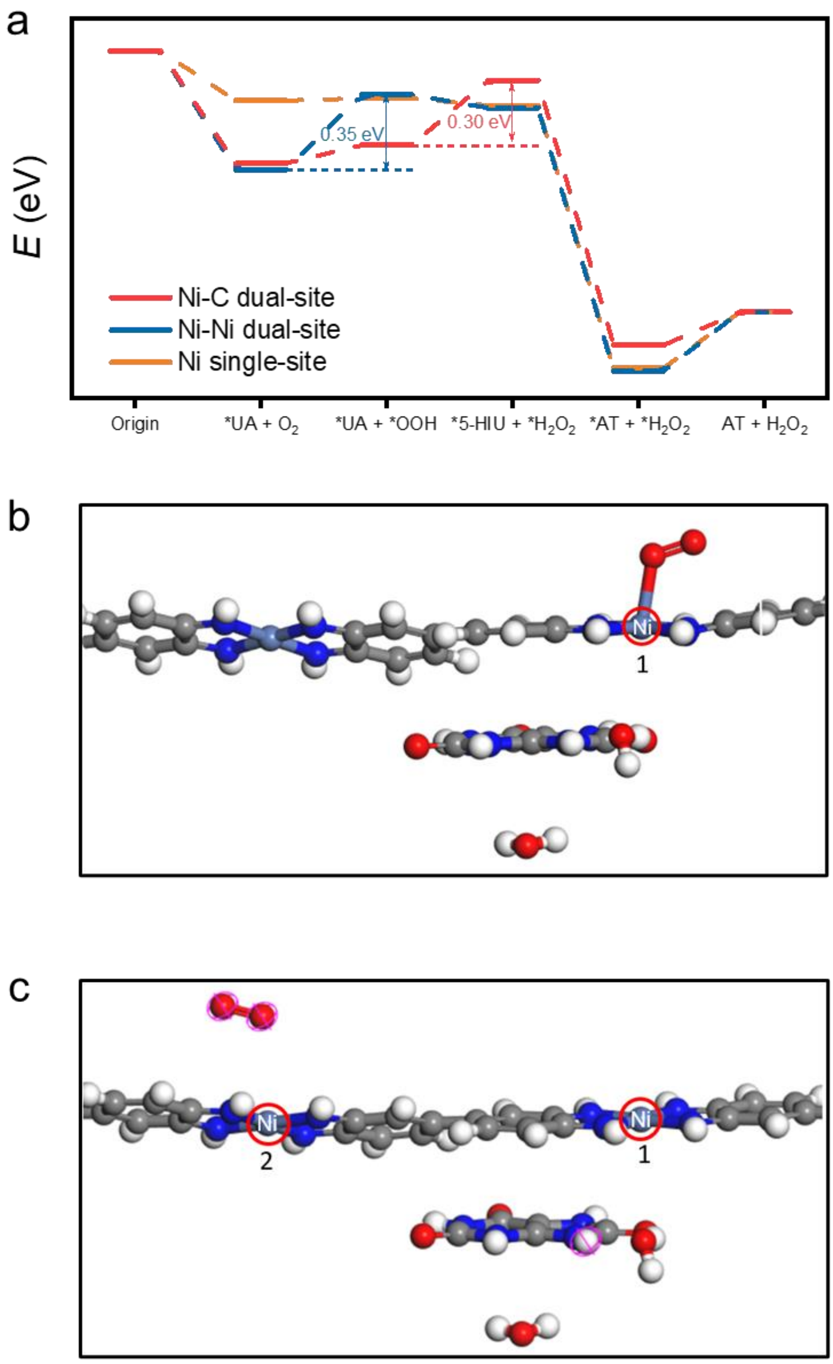

Supplementary Fig. 53. a, Free energy profiles for UA oxidation catalyzed by Ni-DAB with Ni-C dual-site, Ni-Ni dual-site, and Ni single-site. **b,** Diagram for the Ni single-site of Ni-DAB, in which UA and O_2 bind to the same Ni center. **c**, Diagram for the Ni-Ni dual-site of Ni-DAB, in which UA and O_2 bind to adjacent two Ni centers respectively. The white, grey, blue, red, and light blue balls represent H, C, N, O, and Ni atoms, respectively.

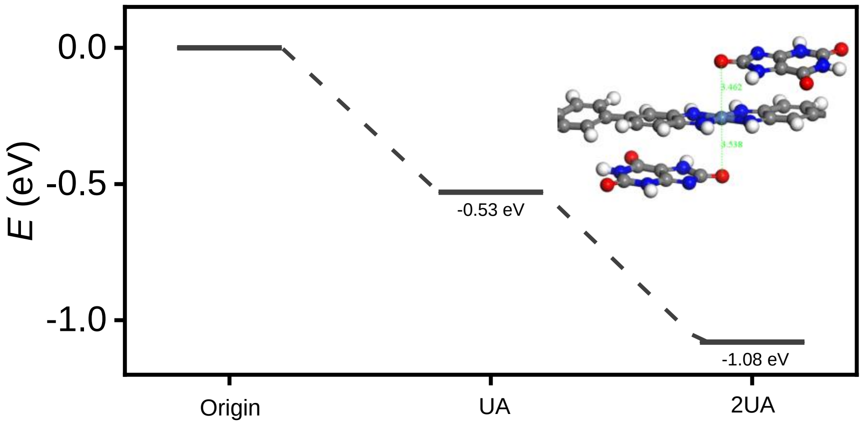

Supplementary Fig. 54. Free energy profile for the adsorption of UA on the same Ni center. The inset shows the structure of two UA adsorbed onto the same Ni center of Ni-DAB. The white, grey, blue, red, and light blue balls represent H, C, N, O, and Ni atoms, respectively.

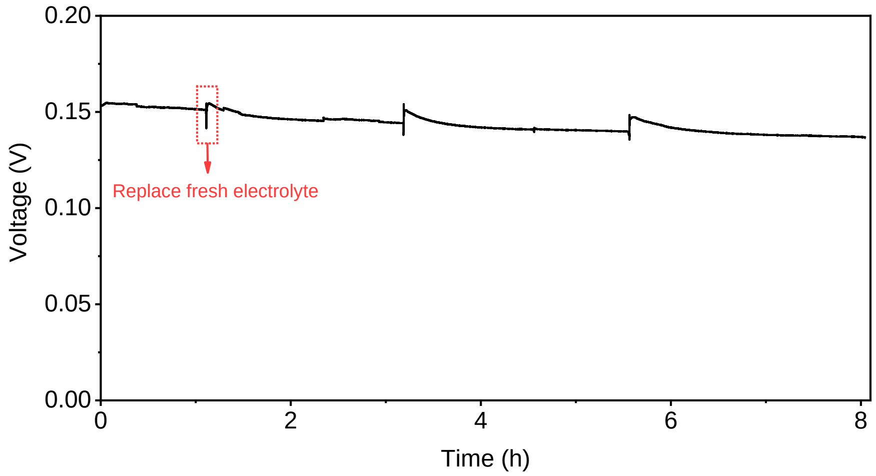

Supplementary Fig. 55. Long-term stability. The stability test of BFC@Ni-DAB/C with 1 kΩ load.

Supplementary Table 1. XPS atomic ratio of Ni-DAB before and after catalytic UA oxidation.

Atomic ratio	C%+N%+Ni%	O%	Cl%
Ni-DAB (before)	92.23	3.93	3.84
Ni-DAB (after)	92.10	7.47	0.43
Variation value		+3.54	-3.41

Supplementary Table 2. Ni K-edge EXAFS curve Fitting Parameters

**Samples**	**path**	***N***	***R* (Å)**	**σ^2 (Å^2)**	**ΔE_0 (eV)**	***R* factor**
Ni foil[b]	Ni-Ni	12	2.48	0.0061	7.63	0.0019
NiPc[c]	Ni-N	4	1.90	0.0017	7.73	0.040
	Ni-C	8	2.92	0.0042		
Ni-DAB[d]	Ni-N	4	1.84	0.0022	2.84	0.019
	Ni-C	4	2.68	0.0034		

[a]*N*, coordination number; *R*, the distance between the absorber and backscatter atoms; σ^2, Debye–Waller factor to account for both thermal and structural disorders; ΔE_0, inner potential correction; *R* factor indicates the goodness of the fit. S_0^2 was fixed to 0.8 as determined from Fe foil fitting. [b]Fitting range: 3.0 ≤ k (/Å) ≤ 13.5 and 1.0 ≤ R (Å) ≤ 3.0. [c]Fitting range: 3.0 ≤ k (/Å) ≤ 12.5 and 1.0 ≤ R (Å) ≤ 3.0. [d]Fitting range: 3.0 ≤ k (/Å) ≤ 12.5 and 1.0 ≤ R (Å) ≤ 3.0.

Supplementary Table 3. Elemental analysis (EA) and inductively coupled plasma mass spectroscopy (ICP-MS) results of Ni-DAB with different amounts of ammonia. The theoretical results were calculated by using an infinite polymerization degree.

Weight ratio	C%	N%	Ni%	H%	O%	Cl%	DAB: Ni
Theoretical results (infinite polymerization degree)	53.59	20.84	21.85	3.72	0	0	1:1
Ni-DAB(3)	46.68	17.62	14.80	3.86	13.44	3.60	1.25:1
Ni-DAB(11)	53.22	19.03	12.85	4.48	7.89	2.53	1.5:1

Supplementary Table 4. Comparison of Michaelis-Menton constants (K_m) and maximum reaction rates (V_{max}) of reported UOX-like biomimetic catalysts.

Samples	K_m (μM)	V_{max} (μM/min)	Refs
pero-nanozysome	16.64	0.00136	12
Co doped MnO_2	22.34	0.00892	13
PRTM-PtNCs	124	9.906	14
MVSM	92.7	15.9	15
Ni-DAB	30	40.8	This work
Ni-DAB/C	28	70.2	This work

Supplementary Table 5. Free energy of stable adsorption of *OOH at different sites

Entry	Site 1: Ni (eV)	Site 2: beta-C (eV)	Site 3: beta-C (eV)
Ni-DAB	0	0.27	-0.18
Co-DAB	0	0.86	0.66

The ball-stick structure is shown in Supplementary Fig. 50. Free energy of the metal center is used as the reference zero.

Supplementary References

1. Delley, B. An all-electron numerical method for solving the local density functional for polyatomic molecules. *J. Chem. Phys.* **92**, 508-517 (1990).
2. Delley, B. From molecules to solids with the DMol3 approach. *J. Chem. Phys.* **113**, 7756-7764 (2000).
3. Perdew, J.P., K. Burke, and M. Ernzerhof Generalized gradient approximation made simple. *Phys. Rev. Lett.* **77**, 3865-3868 (1996).
4. Acharya, C.K. &C.H. Turner CO oxidation with Pt(111) supported on pure and boron-doped carbon: a DFT investigation. *Surf. Sci.* **602**, 3595-3602 (2008).
5. Peng, J.-X., W. Yang et al. Axial coordination regulation of MOF-based single-atom Ni catalysts by halogen atoms for enhanced CO_2 electroreduction. *Nano Res.* **15**, 10063-10069 (2022).
6. Yang, L., X. He, and M. Dincă Triphenylene-bridged trinuclear complexes of Cu: Models for spin interactions in two-dimensional electrically conductive metal–organic frameworks. *J. Am. Chem. Soc.* **141**, 10475-10480 (2019).
7. Lian, Y., W. Yang et al. Unpaired 3d electrons on atomically dispersed cobalt centres in coordination polymers regulate both oxygen reduction reaction (ORR) activity and selectivity for use in zinc–air batteries. *Angew. Chem. Int. Ed.* **59**, 286-294 (2019).
8. Feng, D., T. Lei et al. Robust and conductive two-dimensional metal−organic frameworks with exceptionally high volumetric and areal capacitance. *Nat. Energy* **3**, 30-36 (2018).
9. Park, J., M. Lee et al. Stabilization of hexaaminobenzene in a 2D conductive metal–organic framework for high power sodium storage. *J. Am. Chem. Soc.* **140**, 10315-10323 (2018).
10. Chen, Y., M. Tang et al. A one-dimensional π–d conjugated coordination polymer for sodium storage with catalytic activity in negishi coupling. *Angew. Chem. Int. Ed.* **58**, 14731-14739 (2019).
11. Yang, H.B., S.-F. Hung et al. Atomically dispersed Ni(I) as the active site for electrochemical CO_2 reduction. *Nat. Energy* **3**, 140-147 (2018).
12. Xi, J., R. Zhang et al. A nanozyme-based artificial peroxisome ameliorates hyperuricemia and ischemic stroke. *Adv. Funct. Mater.* **31**, 2007130 (2020).
13. Parmekar, M.V. &A.V. Salker Highly tuned cobalt-doped MnO_2 nanozyme as remarkably efficient uricase mimic. *Appl. Nanosci.* **10**, 317-328 (2019).
14. Liu, Y., Y. Qin et al. Arginine-rich peptide/platinum hybrid colloid nanoparticle cluster: a single nanozyme mimicking multi-enzymatic cascade systems in peroxisome. *J. Colloid Interf. Sci.* **600**, 37-48 (2021).
15. Liu, D., P. Yang et al. Study on performance of mimic uricase and its application in enzyme-free analysis. *Anal. Bioanal. Chem.* **413**, 6571-6580 (2021).
